# Prenatal Alcohol Exposure Impairs the Placenta–Cortex Transcriptomic Signature, Leading to Dysregulation of Angiogenic Pathways

**DOI:** 10.3390/ijms241713484

**Published:** 2023-08-30

**Authors:** Camille Sautreuil, Maryline Lecointre, Céline Derambure, Carole Brasse-Lagnel, Philippe Leroux, Annie Laquerrière, Gaël Nicolas, Sophie Gil, Daniel D. Savage, Stéphane Marret, Florent Marguet, Anthony Falluel-Morel, Bruno J. Gonzalez

**Affiliations:** 1 University Rouen Normandie, INSERM U1245, Team Epigenetics and Pathophysiology of Neurodevelopmental Disorders, 76183 Rouen, France; camille.sautreuil2@univ-rouen.fr (C.S.); maryline.lecointre@univ-rouen.fr (M.L.); carole.lagnel@univ-rouen.fr (C.B.-L.); philippe.leroux@univ-rouen.fr (P.L.); annie.laquerriere@chu-rouen.fr (A.L.); stephane.marret@chu-rouen.fr (S.M.); florent.marguet@chu-rouen.fr (F.M.); anthony.falluel-morel@univ-rouen.fr (A.F.-M.); 2 University Rouen Normandie, INSERM U1245, Team Genetic Predisposition to Cancer, 76000 Rouen, France; celine.derambure1@univ-rouen.fr; 3 Joint Genomics Facilities, Rouen University, 76183 Rouen, France; 4 Department of Pathology, Rouen University Hospital, 76183 Rouen, France; 5 University Rouen Normandie, INSERM U1245, Team Genomics for Brain Disorders, 76183 Rouen, France; gael.nicolas1@univ-rouen.fr; 6 INSERM UMR-S1144, Sorbonne Paris Cité, Université Paris Descartes, 75006 Paris, France; sophie.gil@parisdescartes.fr; 7 Department of Neurosciences, School of Medicine, University of New Mexico, Albuquerque, NM 87131, USA; dsavage@salud.unm.edu; 8 Department of Neonatal Paediatrics and Intensive Care, Rouen University Hospital, University Rouen Normandie and CHU Rouen, 76183 Rouen, France

**Keywords:** FASD, neuroplacentology, neurovascular, neurodevelopment, diagnosis

## Abstract

Although alcohol consumption during pregnancy is a major cause of behavioral and learning disabilities, most FASD infants are late- or even misdiagnosed due to clinician’s difficulties achieving early detection of alcohol-induced neurodevelopmental impairments. Neuroplacentology has emerged as a new field of research focusing on the role of the placenta in fetal brain development. Several studies have reported that prenatal alcohol exposure (PAE) dysregulates a functional placenta–cortex axis, which is involved in the control of angiogenesis and leads to neurovascular-related defects. However, these studies were focused on PlGF, a pro-angiogenic factor. The aim of the present study is to provide the first transcriptomic “placenta–cortex” signature of the effects of PAE on fetal angiogenesis. Whole mouse genome microarrays of paired placentas and cortices were performed to establish the transcriptomic inter-organ “placenta–cortex” signature in control and PAE groups at gestational day 20. Genespring comparison of the control and PAE signatures revealed that 895 and 1501 genes were only detected in one of two placenta–cortex expression profiles, respectively. Gene ontology analysis indicated that 107 of these genes were associated with vascular development, and String protein–protein interaction analysis showed that they were associated with three functional clusters. PANTHER functional classification analysis indicated that “intercellular communication” was a significantly enriched biological process, and 27 genes were encoded for neuroactive ligand/receptors interactors. Protein validation experiments involving Western blot for one ligand–receptor couple (Agt/AGTR1/2) confirmed the transcriptomic data, and Pearson statistical analysis of paired placentas and fetal cortices revealed a negative correlation between placental Atg and cortical AGTR1, which was significantly impacted by PAE. In humans, a comparison of a 38WG control placenta with a 36WG alcohol-exposed placenta revealed low Agt immunolabeling in the syncytiotrophoblast layer of the alcohol case. In conclusion, this study establishes the first transcriptomic placenta–cortex signature of a developing mouse. The data show that PAE markedly unbalances this inter-organ signature; in particular, several ligands and/or receptors involved in the control of angiogenesis. These data support that PAE modifies the existing communication between the two organs and opens new research avenues regarding the impact of placental dysfunction on the neurovascular development of fetuses. Such a signature would present a clinical value for early diagnosis of brain defects in FASD.

## 1. Introduction

Alcohol consumption during pregnancy is a major cause of neurodevelopmental disorders [1]. Based on typical facial features, it is possible for a clinician to diagnose fetal alcohol syndrome (FAS), the more severe expression of fetal alcohol spectrum disorder (FASD) [2]. However, while most FASD infants are devoid of characteristic facial traits, they will gradually develop behavioral and cognitive troubles, such as attention deficit/impulsivity/hyperactivity, speech and language delays, and/or learning disabilities [2]. Consequently, most infants exposed to alcohol in utero are frequently diagnosed at school age and sometimes misdiagnosed [3]. Therefore, several years of clinical care are lost at critical stages of neurodevelopment, when brain plasticity is at its maximum (from birth to 3 years of age).

In terms of epidemiology, several studies have shown that the worldwide prevalence of FASD is estimated to be 7.7 per 1000 people, with strong disparities among countries [4]. In Europe, FAS infants represent 3.7 per 1000 people, while the prevalence of FASD is estimated to be 9- to 10-fold higher [4,5]. Altogether, these data highlight the urgency of developing the prevention of alcohol use during pregnancy and, concurrently, early detection of FASD infants. Whereas maternal self-reporting is frequently used by clinicians as an indication of alcohol consumption during pregnancy, numerous investigations were undertaken to characterize “exposure” biomarkers [6]. These biomarkers are based on the detection of alcohol, its metabolites, as well as different factors, resulting, for example, from hepatic toxicity. Depending on the nature of the “exposure” biomarker, it can be detected in various types of samples, such as maternal or neonatal blood, meconium, or the hair of the babies [6]. Although they can confirm alcohol exposure, these biomarkers do not presume neurodevelopmental defects. In order to overcome this limitation, several research groups have focused on the characterization of “effect” biomarkers of PAE [7,8,9,10]. This generation of biomarkers aims to determine if PAE impacts the central nervous system of the fetus and is likely to alter the neurodevelopmental trajectory during childhood and adolescence.

Over the last decade, neuroplacentology has emerged as a new field of research that focuses on the role of the placenta in brain development [11,12,13]. In particular, it has been evidenced that the placenta is involved in the control of brain angiogenesis and that PAE alters the development of the cortical vasculature [9]. Concurrently, several research groups clearly established that proper brain angiogenesis is a prerequisite for correct neurodevelopment [14,15,16], and recent studies on both pre-clinical models and in humans have revealed that PAE impairs vessel-associated migration and the positioning of two neural cell populations, i.e., GABAergic interneurons [17,18] and oligodendrocytes [19,20]. Altogether, these data have paved the way for the identification of placental biomarkers of neurovascular damage resulting from PAE (patents WO2016207253 and WO2018100143) [21,22].

At a mechanistic level, it has been shown that PlGF, a member of the VEGF family, contributes to vascular development in the fetal brain [9]. Loss of function experiments revealed that the down-regulation of PlGF in placenta resulted in a marked disorganization of the cortical vasculature of the fetus [9]. Consistent with these data, *pgf* knock-out mice displayed a very similar vascular phenotype [23]. Regarding PAE, Savage’s group demonstrated using a rat pre-clinical model in which alcohol reduced the expression of PlGF in the placenta [24], and similar data were also found in human placentas from alcohol-consuming women [9]. Finally, over-expression of the placental *pgf* gene reduced, at least in part, the vascular defects induced by PAE in the fetal brain [9]. Altogether, these data strongly support that PAE affects placenta–cortex communication and, in particular, angiogenic interactions. However, vascular development is a complex process controlled by numerous pro-angiogenic factors and pathways [25]. Although the data obtained for PlGF are promising, a wider approach is required to reinforce this hypothesis.

The aims of the present study were fourfold: (*i*) to establish the first inter-organ “placenta–cortex” transcriptomic signature in mice; (*ii*) to characterize the impact of PAE on the placenta–cortex signature; (*iii*) to identify dysregulated angiogenic factors potentially involved in the placenta–cortex communication; and (*iv*) to validate transcriptomic data at the protein level in one dysregulated ligand–receptor couple.

## 2. Results

Statistical analysis details are provided in Supplementary Table S1.

### 2.1. Transcriptomic Placenta–Cortex Signatures

**Figure 1:**
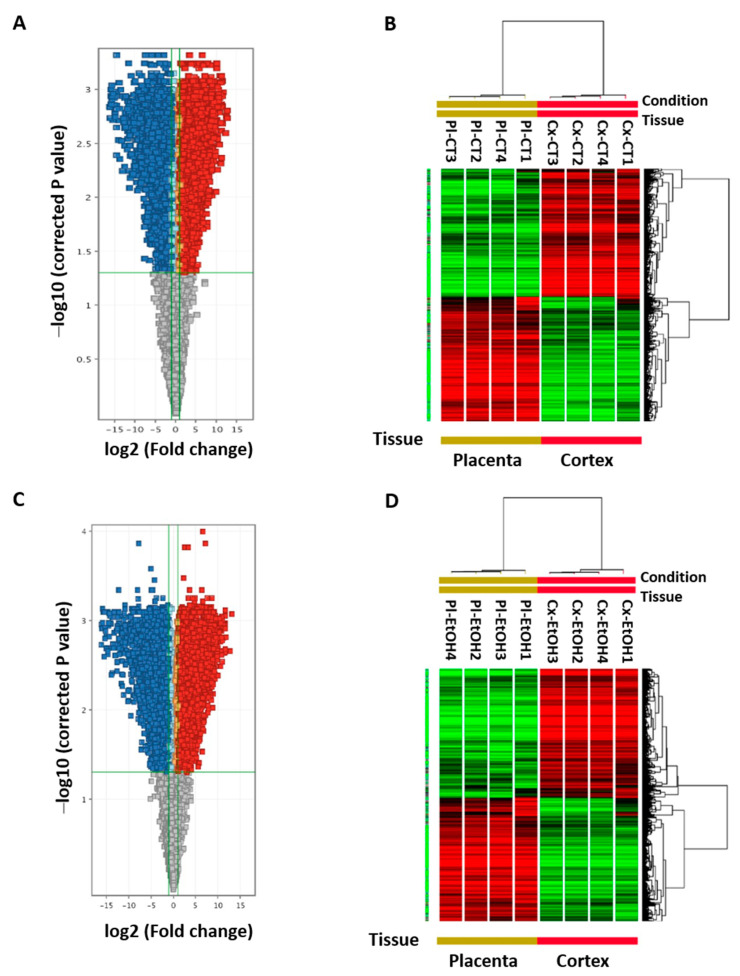
Comparative analysis of microarrays between GD20 placentas and matched fetal cortices in control condition and after alcohol exposure. (**A**,**C**) Volcano plots visualizing genes at least 2-fold under- (blue) or over- (red) expressed between cortex versus placenta at GD20, in control (**A**) and ethanol groups (**C**), after removal of background <50, spikes and control flags. Upon the 26,977 genic probes identified after array extraction in control condition, 6066 and 6238 probes are, respectively under- and over-expressed in the cortex compared to the placenta (**A**). Upon the 27,267 genic probes identified after array extraction in ethanol-exposed conditions, 6326 and 6584 probes are, respectively under- and over-expressed in the cortex compared to the placenta (**C**). (**B**,**D**) Hierarchical clustering of genic probes at least 2-fold under- (red) or over- (green) expressed in the cortex versus the placenta after filtration of microarrays according to Pearson coefficient metric and complete linkage.

For each independent array, mRNA expression profiles were analyzed in five pooled placentas and matching fetal cortices per group. Microarray data extraction with the Feature Extraction Software was submitted to Genespring analysis GX V.12.6 (Figure 1). For control and alcohol groups, respectively, 12,304 and 12,910 genic probes had an absolute fold change ≥ 2 between brain versus placenta and a *p*-value ≤ 0.05. Genic probes are represented in color (blue or red) in Volcano plots (Figure 1A,C). In the control condition, 6066 genes are under- (blue) and 6238 genes are over- (red) expressed in cortex versus placenta (Figure 1A). In the ethanol treatment condition, 6326 genes are under- (blue) and 6584 genes are over- (red) expressed in cortex versus placenta (Figure 1C). Heat maps for these 12,304 and 12,910 genes have been designed with a clustering based on treatment and samples (Figure 1B,D). Detailed lists of genes that are under- or over-expressed between the two organs are given for control and ethanol groups (Supplementary Tables S2 and S3).

### 2.2. Comparison of the Placenta–Cortex Signatures from Control and Alcohol-Exposed Mice

**Figure 2:**
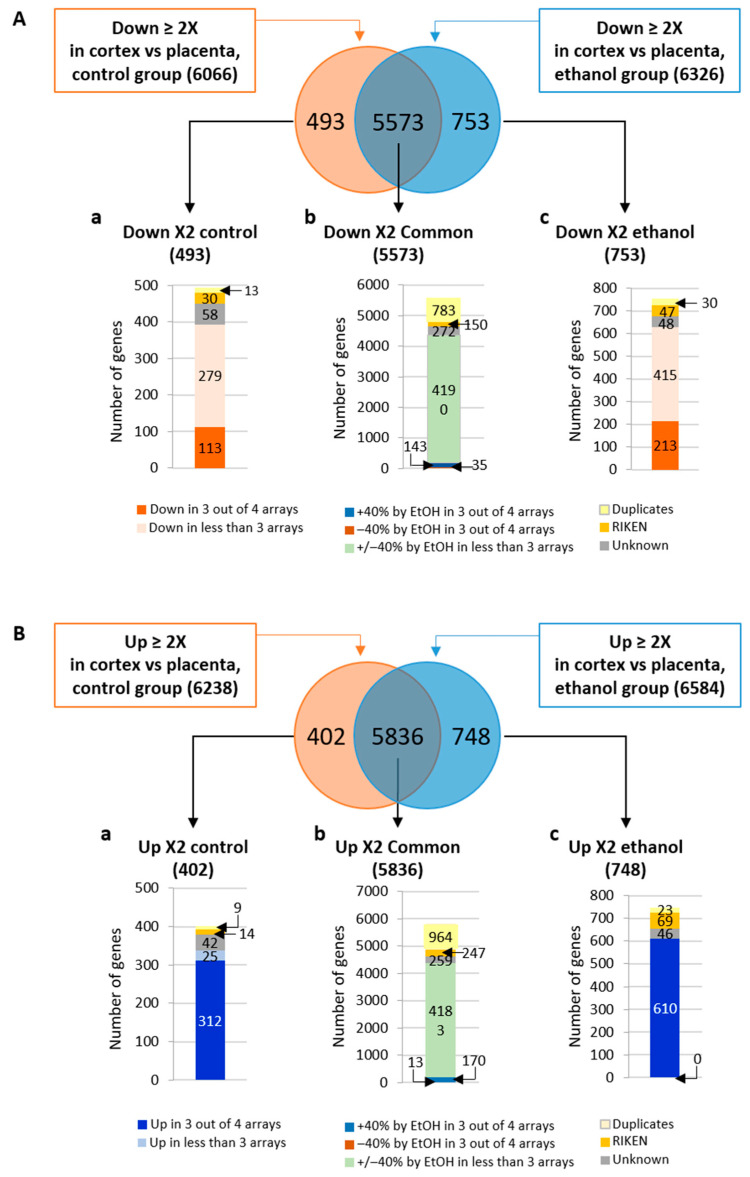
Comparative analysis of the control and alcohol-exposed placenta–cortex signatures. (**A**) Venn diagram showing the number of genic probes being under-expressed in the cortex versus the placenta both in control and alcohol groups (5573), the number of genic probes under-expressed in the cortex versus placenta only in the control signature (493) and the number of genic probes under-expressed in the cortex versus placenta only in the ethanol signature (753). (**B**) Venn diagram showing the number of genic probes being over-expressed in the cortex versus the placenta both in control and alcohol signatures (5836), the number of genic probes over-expressed in the cortex versus placenta only in the control signature (402) and the number of genic probes over-expressed in the cortex versus placenta only in the ethanol signature (748). (**a**–**c**) Histograms below each Venn diagram represent the results from filtration performed on each population. (**a**) Filtration on genes specifically found in the control signatures. (**b**) Filtration on genes commonly found in both signatures. (**c**) Filtration on genes specifically found in the ethanol signatures. For all categories, unknown genes, Riken sequences and duplicates were removed. For control OR ethanol-specific variations, the selection criteria were “observed in at least 3 out of 4 replicates”. For control AND ethanol common variations, the selection criteria were “ethanol induces a 40% change in the cortex/placenta ratio in at least 3 out of 4 replicates”.

A Genespring analysis was performed to establish the list of genes differentially expressed in the placenta/cortex signatures between control and ethanol conditions, and data were represented using a Venn diagram (Figure 2). Results showed that the 12,392 ≥ twofold under-expressed genes in cortex versus placenta are distributed between 493 genes specifically found in the control group, 753 genes unique to the ethanol group and 5573 genes commonly under-expressed in cortex versus placenta for both treatments (Figure 2A). Filtrations were achieved on these gene lists to retain entities which are significantly under-expressed in at least three out of four experiments. In addition, genic probes corresponding to unknown genes, Riken sequences or duplicates were removed (Figure 2A(a–c)). For the 5573 common genes, an additional filtration was based on selecting genes whose cortex/placenta expression ratio was affected by at minimum +/−40% by ethanol in at least three out of four arrays (Figure 2A(b)). These filtration steps resulted in a list of 113 genes under-expressed in brain versus placenta only in the control signature (Figure 2A(a)), 213 genes under-expressed in brain versus placenta only in the ethanol-exposed signature (Figure 2A(c)) and 178 genes under-expressed in cortex versus placenta in both groups, but whose levels were modified by at least 40% under ethanol treatment (Figure 2A(b)). Similarly, a comparative analysis showed that the 12,822 ≥ twofold over-expressed genes in cortex versus placenta are distributed between 402 genes specifically found in the control signature, 748 genes unique to the ethanol signature and 5836 genes commonly over-expressed in cortex versus placenta in both signatures (Figure 2B). The same filtration method was applied and led to a list of 312 genes over-expressed in brain versus placenta only in the control group (Figure 2B(a)), 610 genes over-expressed in brain versus placenta only in the ethanol-exposed group (Figure 2B(c)) and 183 genes over-expressed in cortex versus placenta in both signatures but whose levels were modified by at least 40% under ethanol treatment (Figure 2B(b)). Detailed lists of the control- and ethanol-signature specific genes resulting from the Genespring analysis are presented in Supplementary Tables S4–S7. Results of the filtrations applied to these lists are presented in Supplementary Tables S8–S11. Detailed lists of genes present in the two placenta–cortex signatures and identified from the Genespring analysis are given in Supplementary Tables S12 and S13. The filtration method applied to these lists is presented in Supplementary Tables S14 and S15. A complete Biological Process GO analysis of genes from the placenta–cortex signature dysregulated by PAE was performed and showed, as expected, that several GO terms related to angiogenesis and/or vascular development such as GO:0001955 (blood vessel maturation; fold enrichment 4.25) or GO:1905555 (positive regulation of blood vessel branching; fold enrichment 4.96) were impacted (Supplementary Table S16).

### 2.3. Analysis of the Vascular Biological Function

**Figure 3:**
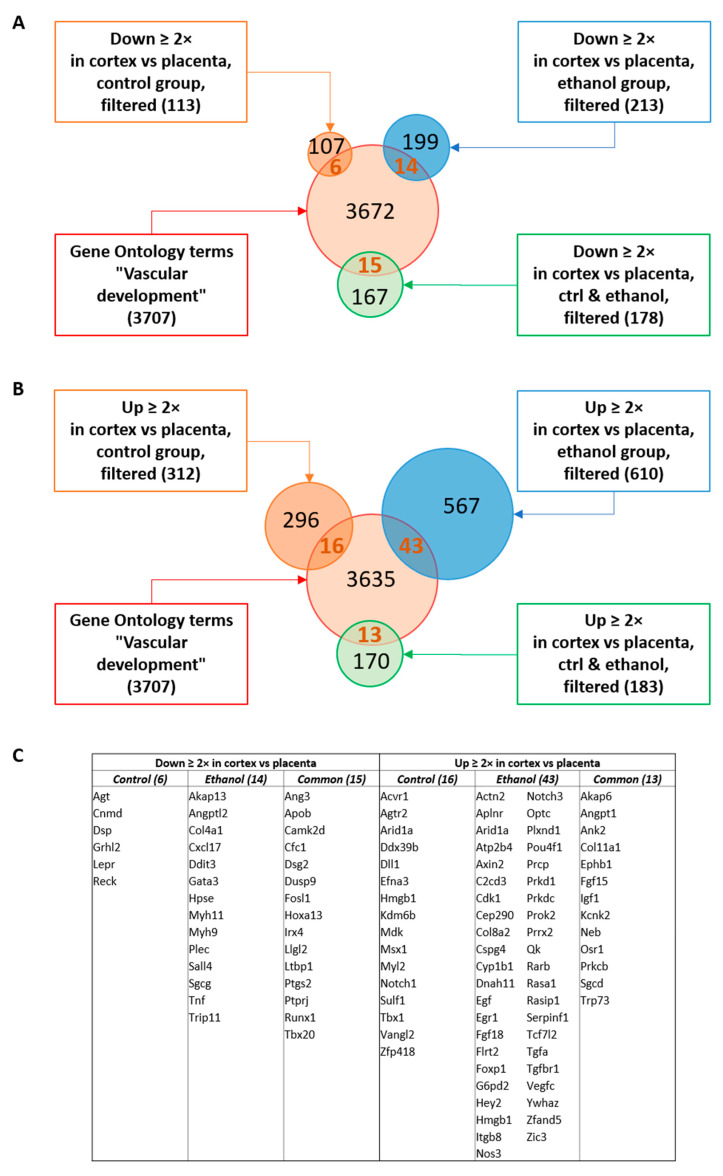
Gene Ontology comparison of the control and alcohol-exposed placenta–cortex signatures with the GO terms of the “vascular development” hierarchy. (**A**) Venn diagram showing the interaction between GO terms “vascular development” (3707) and the genic probes being under-expressed in cortex versus placenta only in the control signature (6), the genic probes under-expressed in cortex versus placenta only in the ethanol signature (14) and in both control and alcohol signatures (15). (**B**) Venn diagram showing the interaction between GO terms “vascular development” (3707) and the genic probes being over-expressed in cortex versus placenta only in the control signature (16), the genic probes over-expressed in cortex versus placenta only in the ethanol signature (43), and in both control and alcohol signatures (13). (**C)** Lists of genic probes resulting from the bioinformatic analysis, representing 107 proteins associated with vascular development. A total of 22 proteins are under- (6) or over- (16) expressed on cortex versus placenta only in the control signature, 57 proteins are under- (14) or over- (43) expressed on the cortex versus placenta only in the alcohol signature and 28 proteins present in both signatures (common) are down (15) or up (13) dysregulated (+/− 40%) by alcohol exposure in the cortex versus placenta.

A functional clustering of genes from the placenta–cortex signature dysregulated by PAE was performed. In particular, because emerging evidence supports that PAE impairs fetal brain angiogenesis by altering the placenta–cortex communication [9], the biological function analysis was performed through the lens of vascular development (Figure 3). To this end, a Venn representation was conducted to look at the interaction between Gene Ontology terms related to “Vascular development” (3707 genes) and the lists of genes under- (Figure 3A) or over-expressed (Figure 3B) in cortex versus placenta and dysregulated by PAE. The number of genes belonging to vascular development GO terms was 6 in the list of genes under-expressed in cortex versus placenta and specific to the control signature, 14 in the list of genes under-expressed in cortex versus placenta and specific to the ethanol signature and 15 in the list of genes commonly under-expressed in cortex versus placenta in the two signatures and dysregulated by PAE (Figure 3A). These numbers were 16 in over-expressed genes in cortex versus placenta specific to control group, 43 in over-expressed genes specific to ethanol and 13 in commonly over-expressed genes dysregulated by PAE (Figure 3B). Altogether, 107 genes present in the placenta–cortex signatures and dysregulated by PAE may potentially be involved in vascular development (Figure 3C). A detailed comparison between genes of the placenta–cortex signature dysregulated by PAE and “Vascular development” GO terms is presented in Supplementary Tables S17 and S18.

**Figure 4:**
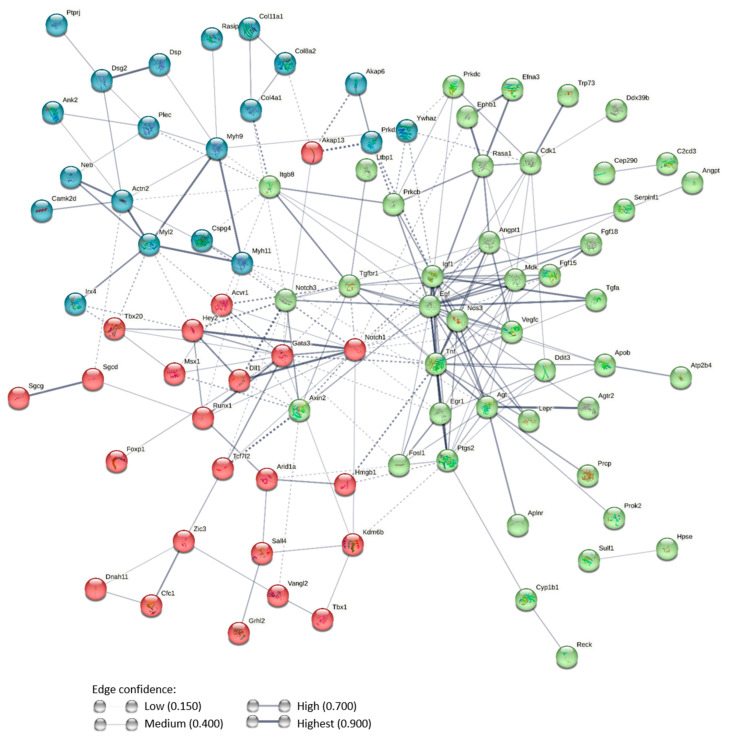
String analysis of genes from the placenta/cortex signature and dysregulated by PAE. The protein–protein interaction (PPI) network was constructed from the 107 proteins identified through the vascular development analysis. The color of nodes is representative of three functional clusters, while the thickness of edges represents the degree of confidence. Solid lines represent PPI within a cluster. Dotted lines represent PPI between clusters.

**Figure 5:**
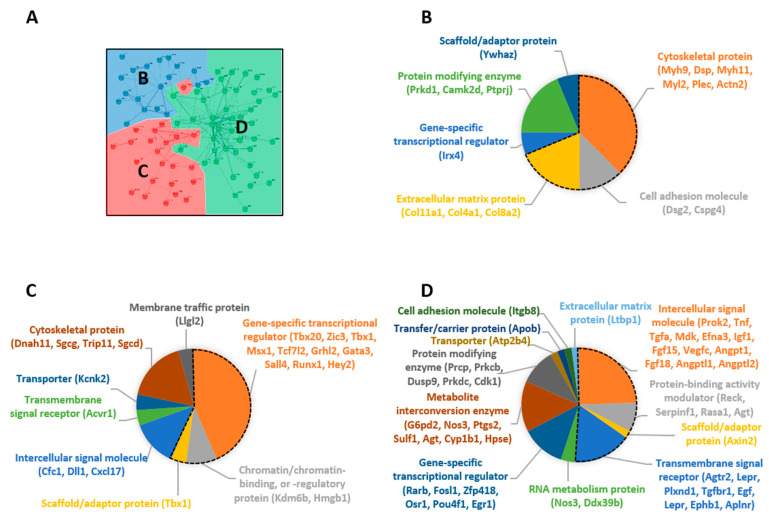
PANTHER functional classification of String clusters by protein class. Annotation and functional classification of proteins belonging to each String cluster was performed using the PANTHER Classification System (Protein ANalysis THrough Evolutionary Relationships; www.pantherdb.org (accessed on 17 January 2023) version 17.0). (**A**) Color map visualizing each cluster submitted to *PANTHER* analysis. (**B**) Among the 16 assigned proteins of the blue-node cluster (20 proteins), more than two-thirds (11 proteins) are involved in cell structure and cell adhesion (dotted black line). (**C**) Among the 23 assigned proteins of the red-node cluster (28 proteins), 13 are regulators of transcriptional activity (dotted black line). (**D**) Among the 34 assigned proteins of the green-node cluster (42 proteins), 25 are intercellular communication molecules (signal molecules or transmembrane receptors; dotted black line). Among the 3 clusters, 14 ligands and 13 receptors have been identified and constitute candidates representative of a PAE-dysregulated placenta–cortex communication.

These 107 intersecting genes were imported into the String biological database for analysis (https://string-db.org/ (accessed on 16 January 2023)). Results provided scores of predicted protein–protein interactions (PPI) and functional clusters (Figure 4). The statistical analysis revealed a significant PPI enrichment with 228 identified interactions (*p* < 1.0 × 10^−16^ versus a random set of proteins of the same size and degree distribution drawn from the genome), implying that 84 proteins among the 107 are biologically connected as a group that can be categorized in three functional clusters (Figure 4). Each cluster was further analyzed with PANTHER 17.0 functional classification tool which allowed us to search for enrichment according to the Gene Ontology domain [protein class] (Figure 5) [26]. The blue-node cluster contains 20 proteins. Six were not functionally assigned. Among the 16 assigned proteins, 11 are involved in cell structure and cell adhesion (cytoskeletal proteins, extracellular matrix proteins, cell adhesion molecules; Figure 5A,B). The red-node cluster contains 28 proteins. Five were not functionally assigned. Among the 23 assigned proteins, 13 are regulators of transcriptional activity (transcription factors, chromatin-binding regulators; Figure 5C). The green node contains the highest number of proteins. Eight were not functionally assigned. Among the 34 assigned proteins, 25 are intercellular communication molecules (signal molecules or transmembrane receptors; Figure 5D). Based on the most enriched components (statistically and in terms of protein numbers), each cluster was tagged as follows: (i) structural proteins and regulation (blue cluster; Table 1), (ii) transcriptional regulation (red cluster; Table 1), and (iii) intercellular communication (green cluster; Table 1).

The three clusters were also classified using the *PANTHER* Overrepresentation Test, according to the GO-slim domain [biological process] (Table 1). A significant enrichment rate was detected in the transcriptional regulation cluster (red cluster) for biological processes related to morphogenesis, including nervous system development (Table 1, column 1). A significant enrichment rate was detected in the intercellular communication cluster (green cluster) for biological processes related to cell proliferation, migration, axon guidance and angiogenesis (Table 1, column 2). Finally, a significant enrichment rate was detected in the structural proteins and regulation cluster (blue cluster) for a unique biological process: muscle cell differentiation. (Table 1, column 3). Detailed arborescence of biological processes provided via the PANTHER analysis is provided for each cluster in Supplementary Tables S19–S21.

Considering that the PANTHER functional classification revealed a marked enrichment of the intercellular communication category, we further analyzed this functional cluster. Interestingly, among the 107 genes submitted to clustering, 27 (more than 25%) are described as neuroactive ligand/receptor interactors and 19 belong to the intercellular communication cluster. These genes have been regrouped in Table 2, which represents placental ligands with their matched cortical receptors (top section), and cortical ligands with their matched placental receptors (bottom section), which are either under- or over-expressed in the cortex versus placenta depending on the experimental conditions. These ligand/receptor couples have been classified based on the results from the bioinformatic analysis, according to the organ and the treatment in which the transcript was over/under-expressed. For most ligand/receptor couples, this representation revealed that either the ligand or the receptor was × differentially expressed in one organ versus the other organ, and this differential expression pattern was disrupted following alcohol exposure. When only one member of the couple ligand/receptor fulfilled all filtration analysis criteria, we indicated in light characters the information regarding its respective partner(s) (% variation under PAE within n replicates out of four). Interestingly, the couple, Agt/Agtr2, presents an over-expression of the ligand in the control placenta, which is not seen after ethanol treatment, associated with a significant over-expression of the receptor in the control brain, which also disappeared after ethanol exposure. Altogether, these data strongly support that ligands and/or receptors from the intercellular communication cluster constitute candidates reflecting a placenta–cortex communication dysregulated by PAE.

### 2.4. Molecular Validation of a Selected Ligand/Receptor Couple

**Figure 6:**
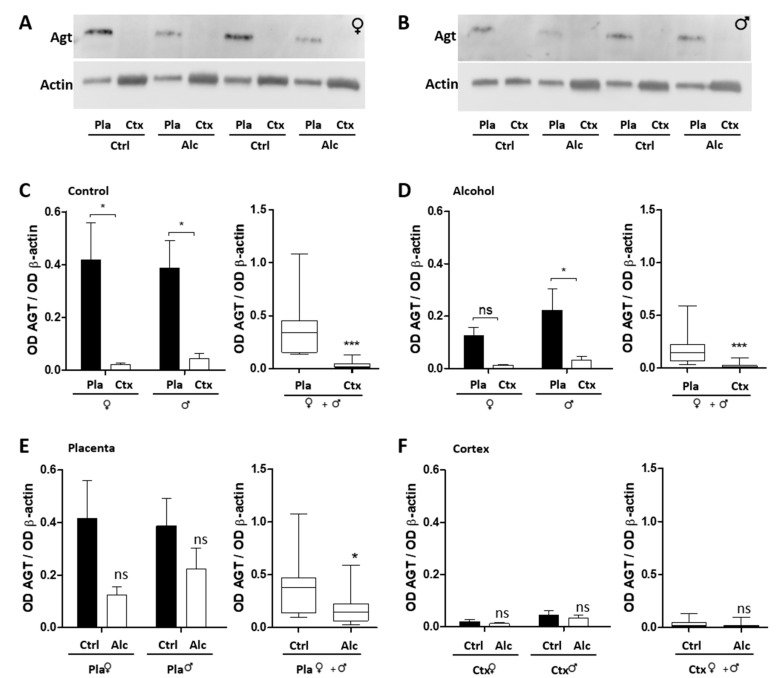
Protein validation of angiotensinogen expression in paired placenta/cortex extracts from control and alcohol-exposed mice. (**A**,**B**) Western blots visualizing the relative expression of angiotensinogen in placentas and paired cortices of females (**A**) and males (**B**) fetuses from control and alcohol-exposed mice. (**C**) Quantification of the relative placenta/cortex expression of angiotensinogen in females and males of the control group. *, *p* < 0.05; ***, *p* < 0.001 vs. Placenta. (**D**) Quantification of the relative placenta/cortex expression of angiotensinogen in females and males of the PAE group. *, *p* < 0.05; ***, *p* < 0.001 vs. Placenta. (**E**) Comparison of angiotensinogen expression in female and male placentas of control and PAE mice. *, *p* < 0.05 vs. Control. (**F**) Comparison of angiotensinogen expression in female and male cortices of control and PAE fetuses.

As a first step to validate bioinformatic results regarding ligand/receptor candidates potentially dysregulated in the placenta–cortex signature, we selected the angiotensinogen/AGTR1/2 receptors pairsm since both ligand and AGTR2 were affected by PAE. The PAE model has been repeated on new pregnant mice, and brain and matched placenta extracted and processed for Western blot analysis. In agreement with the microarray analysis, the angiotensinogen protein (Agt) is preferentially detected in placental extracts (Figure 6A,B). In control condition, quantifications showed that Agt levels are significantly higher in placenta than cortex in both male and female embryos (*p* < 0.05, Figure 6C). In the presence of alcohol, a significant difference between organs is observed only in males and when pooling all embryos (Figure 6D). The comparison of Agt levels in placenta and cortex in the absence of and after PAE revealed a tendency toward a decrease in Agt in both organs, but this effect was significant only in placenta and when pooling female and male embryos (Figure 6E,F).

**Figure 7:**
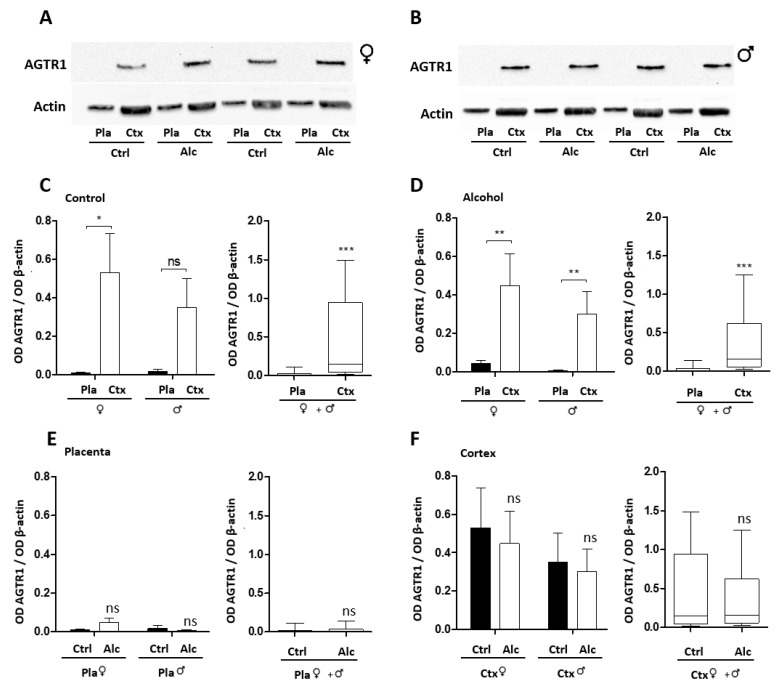
Protein validation of AGTR1 receptor expression in paired placenta/cortex extracts from control and alcohol-exposed mice. (**A**,**B**) Western blots visualizing the relative expression of AGTR1 in placentas and paired cortices of female (**A**) and male (**B**) fetuses from control and alcohol-exposed mice. (**C**) Quantification of the relative placenta/cortex expression of AGTR1 in females and males in the control group. *, *p* < 0.05; ***, *p* < 0.001 vs. Placenta. (**D**) Quantification of the relative placenta/cortex expression of AGTR1 in females and males in the PAE group. **, *p* < 0.01; ***, *p* < 0.001 vs. Placenta. (**E**) Comparison of AGTR1 expression in female and male placentas in control and PAE mice. (**F**) Comparison of AGTR1 expression in female and male cortices of control and PAE fetuses.

Regarding receptors, AGTR1 is preferentially detected in the cortex for both genders (Figure 7A,B). In the control group, AGTR1 is significantly over-expressed in the cortex of female embryos (*p* < 0.05) and pooled embryos (*p* < 0.001; Figure 7C). In the PAE group, AGTR1 is also significantly more expressed in the cortex than in the placenta in females and males (*p* < 0.01) and pooled embryos (*p* < 0.001; Figure 7D). However, for a given organ (placenta or cortex), the effect of PAE on AGTR1 levels is not significant for both genders (Figure 7E,F). The second angiotensinogen receptor, AGTR2, was mainly detected in placenta via Western blot. The effect of PAE on this receptor was similar to that of AGTR1. Data concerning this receptor are presented in Supplementary Figure S1.

**Figure 8:**
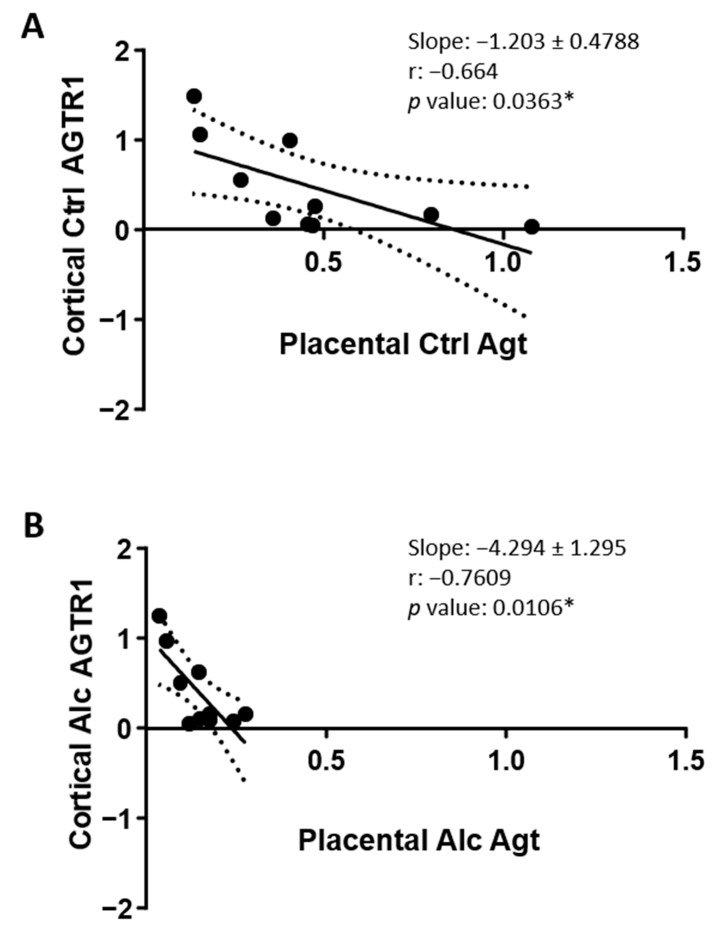
Pearson correlation analysis between placental AGT expression and cortical AGTR1 expression. (**A**) Graph visualizing the paired expression of placental AGT and cortical AGTR1 in the control group. (**B**) Graph visualizing the paired expression of placental AGT and cortical AGTR1 in the PAE group. *, *p* < 0.05.

Taking advantage of the fact that for all fetuses, placentas and cortices are paired, a Pearson analysis has been conducted in order to research a correlation between placental Agt and cortical AGTR1 (Figure 8). In the control group, analysis revealed a significant negative correlation: the cortical expression of AGTR1 decreases when the placental level of Agt increases (slope = −1.203 ± 0.4788). The r value of the linear regression is −0.664, *p* = 0.0363* (Figure 8A). In the ethanol-exposed group, the slope of the linear regression drastically decreases towards more negative values (−4.294 ± 1.295) with an r value of −0.7609, *p* = 0.0106*, confirming that placental Agt is decreased under alcohol exposure, and indicating that lowest Agt levels in the placenta correlate with the highest AGT1R levels in the cortex (Figure 8B). A similar Pearson analysis has been realized concerning placental Agt and placental AGTR2 (Supplementary Figure S2). Interestingly, Agt and AGTR2 levels display a significant correlation in the alcohol group only (slope −14.91 ± 6.05; r = −0.6571; *p* = 0.039*), supporting that PAE triggered a regulatory mechanism which was unseen in the control condition (*p* = 0.4724, ns; Supplementary Figure S2).

### 2.5. Comparison of Angiotensinogen Immunolabeling in the Placenta of a Control and Alcohol-Exposed Human Case

**Figure 9:**
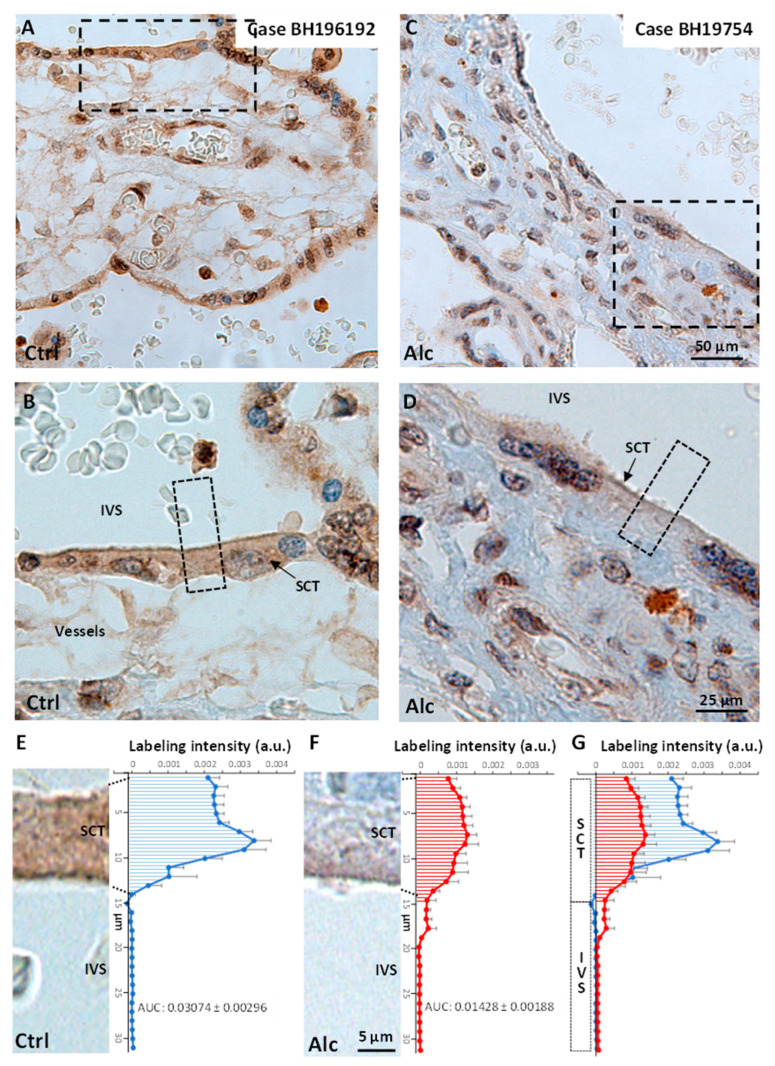
Angiotensinogen immunoreactivity in human placentas from control and alcohol-exposed cases. (**A**,**B**) Visualization at low (**A**) and high (**B**) magnifications of angiotensinogen immunolabeling in villi of a control case. (**C**,**D**) Visualization at low (**C**) and high (**D**) magnifications of angiotensinogen immunolabeling in placental villi from an alcohol-consuming woman. (**E**,**F**) Scanline analysis of the intensity profile of angiotensinogen immunolabeling in the syncytiotrophoblastic layer of a control case (**E**) and an alcohol-consuming woman (**F**). (**G**) Overlay of the two scanline intensity profiles. Hatched areas represent the integrated zone used to measure the area under the curves (AUC). SCT: syncytiotrophoblast; IVS: Intervillous space. Dotted rectangles visualize regions shown at higher magnification.

The expression profiles of angiotensinogen immunoreactivity were analyzed in 38WG control and 36WG alcohol-exposed placentas (Figure 9). As previously shown in the literature [27], Agt immunolabeling was detected in the control case at the level of the syncytiotrophoblasts (SCT) and cytotrophoblasts (Figure 9A,B). A scanline analysis was performed to visualize the Agt intensity profile in the SCT and the intervillous space (IVS) after normalization with internal background (Figure 9E). Data indicated that the mean integrated value of immunoreactive signal in the SCT thickness was 0.03074 ± 0.0023 AU² (Figure 9E). In the alcohol case, the thickness of the SCT was similar to that of the control case (Figure 9C,D) and Agt immunolabeling was also detected in the SCT (Figure 9C,D). However, the scanline analysis indicated that the normalized Agt intensity profile was lower (Figure 9F,G)**.** Because these observations were related to a unique alcohol-exposed case report, no statistical analysis was performed.

## 3. Discussion

### 3.1. Inter-Organ versus Intra-Organ Transcriptomic Studies

To date, several transcriptomic [24,28,29], proteomic [30] and epigenetic [31] studies investigated the effects of PAE on different organs, including the brain [29] and placenta [24]. In the fetal brain, a transcriptomic study from Hashimoto-Torii and co-workers compared the effects of PAE on gene expression in human and mouse cortices [29]. The authors identified several enriched biological pathways after PAE, including Notch and VEGF signaling, and correlated them with neurodevelopmental processes. Similarly, a study performed in the mouse rostro-ventral neural tube investigated the effect of PAE and evidenced enrichment of biological functions such as cell migration [28]. Regarding rat placenta, a microarray analysis from Rosenberg and co-workers showed that alcohol consumption during pregnancy induced a significant alteration in the expression of 22 genes, including matrix metalloproteinases and PlGF [24]. Interestingly, these studies identified genes belonging to biological processes such as vascular and nervous system development. However, all these studies have a commonality: they all involve intra-organ analyses. In other words, the authors investigated the effects of PAE on the expressome of a given organ. Although essential to characterize the effect of a given treatment on a given organ, this strategy would be less powerful when unveiling altered communication between two organs such as the placenta and the brain. In this sense, intra-organ studies would be less efficient at identifying imbalanced ligand/receptor couples when the ligand and/or its receptor(s) are expressed and dysregulated at a distance. The present study provides the first inter-organ transcriptomic placenta/cortex signature of PAE. Here, we hypothesized that the placenta and fetal brain communicate through circulating factors and that comparison of control and PAE inter-organ signatures may reveal the effects of alcohol on secretory/hormonal functions. This was the rationale for carrying out two inter-organ signatures (control and PAE), followed by a subsequent comparison of these signatures. In the control group, microarray analysis demonstrated that 12,304 genes were differently expressed between the developing fetal cortex and the placenta at GD20. Interestingly, whereas for most genes, alcohol did not modify the placenta/cortex transcriptomic profile, a comparison of the two control and PAE signatures revealed that 1248 genes were exclusively present in only one of the two signatures, indicating that, for these genes, a marked imbalance occurred after PAE. Altogether, these data showed that PAE modified the placenta/cortex transcriptomic signature and raised the question of functional processes linking together these 1248 genes.

### 3.2. Functional Clustering of Dysregulated Angiogenic Genes from the Placenta–Cortex Signature

Both clinical and pre-clinical studies have established that alcohol impacts angiogenesis in adults and during development [19,32,33,34,35,36]. For example, in adult rats, in vivo experiments showed that acute ethanol exposure impairs wound healing by inhibiting angiogenesis [33]. In adult non-human primates, alcohol consumption decreased endothelial progenitor cells’ ability to form new capillaries [34]. In humans, chronic moderate alcohol consumption has been shown to stimulate angiogenesis, potentially contributing to the progression and metastasis of cancers [37]. During development, several studies evidenced that alcohol consumption during pregnancy is able to impair angiogenesis in different organs, including the brain [35], retina [36] and placenta [9,32]. The first arguments in favor of a placental contribution in the control of brain angiogenesis resulted from studies using animal models [9]. Indeed, a targeted repression of the *pgf* gene in mouse placenta mimicked some effects of PAE on the disorganization of cortical microvessels described in the fetal brain, while rescue experiments consisting of a targeted placental over-expression of *pgf* prevented some deleterious effects of PAE on the fetal brain vasculature [9]. Taken together, these results support that PAE, by altering the expression of angiogenic factors, impairs the placenta–cortex connection involved in the control of vascular development. However, whereas PlGF appeared to contribute to the deleterious effects of PAE on fetal brain angiogenesis, no data were available regarding other angiogenic factors and families. The present study provides the first angiogenic screening of the placenta–cortex signatures and reveals that 107 genes of the inter-organ signature involved in vascular development are markedly dysregulated by PAE. In particular, three interaction clusters were identified based on significant functional interactions and tagged according to their main cellular component composition. The first two clusters out of three were tagged “Structural proteins” and “Transcriptional regulation”. Interestingly, the third cluster, which is the more important in terms of protein number, was tagged “Intercellular communication”. This cluster is mostly composed of ligands and receptors, including angiotensinogen, VEGFc, Egf, Fgf angiopoietin, Igf, agtr2, Lepr, Tgfbr1, and Rarb. Altogether, these data indicate that among the PAE-dysregulated genes from the placenta–cortex signature, ligand and/or receptors are markedly enriched, reinforcing the hypothesis of functional communication between these two organs.

### 3.3. Placenta–Cortex Signature and the Example of PlGF

Previous intra-organ transcriptomic [24] and protein [9] studies showed that the expression of PlGF (*pgf* gene) is dysregulated by PAE in both human and rodent placentas. PlGF is a member of the VEGF family which is strongly expressed in the placenta but poorly detected in the fetal brain [38]. In the present inter-organ study, *pgf* is neither detected in the control nor PAE placenta–cortex signatures. In fact, *pgf* is present in the pool of common genes between the two signatures. These inter-organ data are not in contradiction with the previous intra-organ studies [9,24]. Indeed, the present results indicate that, in the control placenta–cortex signature, the expression of *pgf* is at least two-fold lower in the cortex versus placenta and that, in the PAE signature, the cortical expression of *pgf* remains at least two-fold lower in the cortex versus placenta. However, consistent with previous intra-organ studies [9,24], PAE effectively reduced *pgf* expression by 13 to 30% three times out of four (Supplementary Table S13). Altogether, the example of PLGF/*pgf* suggests that genes detected in only one of the two signatures have been robustly impacted by PAE.

### 3.4. Protein Validation of the Angiotensinogen/AT1/2 Receptor Couple

Omics approaches including transcriptomics constitute an exceptional source of big data with a promising value to decipher processes involved in a given pathology. However, a key challenge in the acceptance of transcriptomics-based data is validation [39]. In the present study, bioinformatics analysis generated a list of ligands and/or receptors dysregulated by PAE between the placenta and brain. In order to reinforce the validity of this list, we performed a protein validation of the dysregulated couple Agt, AGTR1 and AGTR2. Validation was performed at three levels. First, Western blot experiments confirmed that, as found by microarrays, Agt, AGTR1 and AGTR2 were differently expressed between the placenta and brain. In particular, in the control signature, Agt was much more expressed in the placenta than in the fetal cortex whereas, on the contrary, AGTR1 was mainly detected in the fetal cortex. Second, intra-organ analysis showed that, even if PAE tended to decrease the expression of Agt in the placenta, the effect of alcohol was not significant except when placentas from female and male fetuses were pooled. Similarly, even if PAE tended to decrease the expression of AGTR1 in the fetal cortex, the effect was not significant. Third, taking advantage of the fact that a given placenta was systematically paired with its associated fetal cortex, correlation analyses revealed that the higher the Agt level in placenta, the more cortical AGTR1 expression was reduced. Interestingly, when PAE decreased Agt expression in placenta, the cortical expression of AGTR1 was also impaired, resulting in a persisting placenta–cortex correlation. Taken together, these data support that placental Agt and cortical AGTR1 expressions are linked and that this ligand/receptor couple may represent a good candidate for a functional validation. These data also suggest that an intra-organ analysis alone may have not been powerful enough to identify Agt as a possible ligand involved in the placenta–cortex communication. They also support that the characterization of unique molecular biomarker of alcohol-induced neurodevelopmental defects is probably unrealistic and that characterization of a multifactorial signature would be probably more promising, as recently proposed for early diagnosis of cancers [40].

### 3.5. Neurodevelopmental Consequences of the Angiogenic Placenta–Cortex Dysfunction

In the last decade, it has been demonstrated in both human and mouse fetuses that (i) in utero alcohol exposure impairs angiogenesis and disorganizes the cortical microvasculature [35] and (ii) PlGF is involved in the control of cortical vascular development [9,23]. Moreover, it is now established that brain microvessels constitute guidance supports for several types of migrating nervous cells, i.e., interneurons [14] and oligodendrocytes [15]. Recently, it has also been shown that immature astrocytes use microvessels as guides during migration in the developing cortex [41]. Consequently, it is tempting to speculate that the alcohol-induced microvascular impairments evidenced in the developing cortex would contribute, in part, to the alcohol-induced neurodevelopmental disorders. Consistent with this hypothesis, our research group recently demonstrated that, in mice, PAE impairs the vessel-associated migration and the positioning of GABAergic interneurons [17] and induces interneuropathies in FAS human fetuses [18]. Similarly, it has also been shown that PAE alters the differentiation of oligodendrocytes migrating along microvessels in both mice and humans [19,20,42]. Altogether, these data support that the placenta, by releasing pro-angiogenic factors, could constitute a promising source of circulating biomarkers for the early diagnosis of vascular-related neurodevelopmental disorders of FASD infants (WO2016207253 and WO2018100143) [21,22].

### 3.6. Placenta Dysfunction and Brain Development, a Growing Concept with Clinical Perspectives

It has been described for long time that the placenta plays a pivotal role in the growth and development of the fetus by providing nutrients, oxygen, removing waste products and also by constituting a protective and selective barrier for the passage of hormones, neurotransmitters, toxic agents and infectious microorganisms. More recently, growing evidence supported a communication between the placenta and fetal brain, and neuroplacentology has emerged as a new field of research focusing on the role of the placenta in brain development [10,11,12,13]. As an example, a placenta–cortex axis involving the expression of LIF by trophoblasts and the resulting induction of cortical Igf has been shown to contribute to the proliferation of neural progenitors in the developing rat brain [43]. Interestingly, Igf1 was one of the ligands which appeared to be dysregulated by PAE in the present placenta–cortex signature. In relation to neuroplacentology, a recent clinical study aimed to establish a transcriptomic placental signature of neonates who developed cerebral white matter damage [44]. The results showed that in the group of infants with white matter lesions, several processes such as inflammation were dysregulated in the placenta [44]. However, due to the difficulty of obtaining brain tissues from human neonates, such clinical studies fail to demonstrate functional/mechanistic links. In the present pre-clinical study, all samples were paired and a given placenta was systematically associated with its fetal brain. This major point of the study allowed the implementation of correlation studies between a given placental ligand (Agt) and its cortical receptor (AGTR1). Such correlation studies should be performed for all of the 27 ligand/receptor candidates identified.

In conclusion, the emergence of neuroplacentology supports that the placenta and the fetus closely interact to control processes such as brain vascular development. The present study provides the first placenta–cortex transcriptomic signature in mice, and it also reveals that PAE markedly impaired the inter-organ signature of several ligands and/or receptors, reinforcing the notion of a communication between the two organs. Among new research avenues, a functional validation by placental gene repression/over-expression associated with analysis of cortical neurodevelopmental defects has to be engaged in order to establish the first placental multifactorial signature of PAE-induced neurovascular impairments. In addition, the present study focused on vascular development, a process markedly altered by PAE. However, a whole biological process analysis of the dataset would provide a unique opportunity to identify other physiological functions dysregulated by PAE and involved in placenta–brain communication. Ultimately, such a signature would have a clinical value for the early diagnosis of FASD.

## 4. Materials and Methods

### 4.1. In Vivo Treatment of Pregnant Mice

National Marine Research Institute (NMRI) mice from Janvier (Le Genest-Saint-Isle, France) were used according to the recommendations of the French Ethical Committee and to the recommendations of the European Communities Council directives (2010/63/UE). Experiments were performed under the supervision of authorized investigators (B.J.G., authorization n°7687 from the Ministère de l’Agriculture et de la Pêche). From gestational day (GD)15 to GD20, twelve pregnant mice received a daily subcutaneous injection of sodium chloride (NaCl 9‰) or alcohol (3 g/kg, Fisher Scientific) diluted in NaCl (50%, *v*/*v*). At GD20, placentas and paired fetal cortices were collected, immediately frozen in liquid nitrogen and stored at −80 °C until use.

### 4.2. RNA Sample Preparation

**Figure 10:**
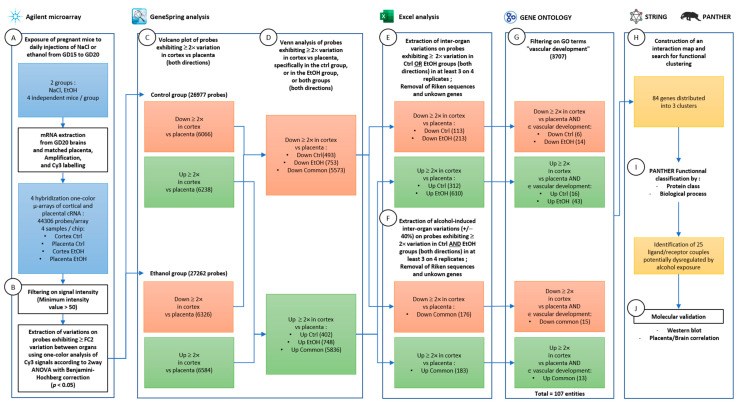
Experimental design of the study. (**A**) The in utero alcohol exposure model consisted of daily *sc* injection of 0.9% NaCl or ethanol (3 g/kg) in pregnant mice from gestational day (GD) 15 to GD20. Each group comprised 4 independent replicates. At GD20, fetuses were collected with their matched placenta. mRNA were extracted from cortices and placentas, amplified and labeled with Cy3. Resulting cRNA were hybridized on Agilent micro-array chips. (**B**) Detected signals were filtered based upon spots intensities and fold change variations between cortex versus placenta. (**C**) Volcano plots were generated using Genespring analysis to sort the genic probes that display, at least, a twofold variation (upper or lower) between cortex and placenta in the control group and after ethanol treatment. (**D)** Comparison of the two (control and alcohol) placenta–cortex signatures. A Venn analysis was carried out to search for genic probes present either in the control placenta–cortex signature, the ethanol-exposed placenta–cortex signature, or both. (**E**) Treatment-specific inter-organ lists were filtered to retain variations in at least 3 out of 4 replicates. (**F**) Genes common in the two placenta–cortex signatures were filtered based on a 40% change in the cortex/placenta ratio after ethanol treatment. (**G**) Entity lists were compared with Gene Ontology “vascular development” terms. (**H**) Resulting gene list was submitted to String analysis for network and functional clustering. (**I**) Proteins within each cluster were analyzed by PANTHER upon cell component and biological process classification. Analysis led to the identification of ligand/receptor couples dysregulated by PAE. (**J**) Protein validation of a PAE-dysregulated ligand/receptor couple by Western blot and placenta/cortex correlation analysis.

According to the manufacturer’s recommendations, placentas from control and alcohol-exposed mice and cerebral cortices from matched fetuses were harvested for total RNA extraction using the NucleoSpin^®^ RNA plus (Macherey-Nagel, Hoerdt, France). The quality and quantity of isolated mRNAs were assessed using the 2100 Bioanalyzer (Agilent Technologies, Santa Clara, CA, USA) and the Nanodrop device (Thermo Scientific, Wilmington, DE, USA). Only RNA samples with a minimal RNA integrity number of 7 were used for subsequent experiments. Pooled samples for microarray study were created with 5 µg of RNA extracted from five control placentas or cerebral cortices and five alcohol-exposed placentas or cerebral cortices at GD20. Matching between a given placenta and its associated fetal cortex was systematically respected. A statistical threshold of four independent experiments corresponding to 16 arrays was fixed. The experimental design of a typical experiment was as follows: 1 pool of 5 control placentas, 1 pool of 5 matching control cerebral cortices, 1 pool of 5 alcohol-exposed placentas and 1 pool of 5 matching alcohol-exposed cerebral cortices (Figure 10A). This design was replicated in four independent experiments.

### 4.3. Transcriptome Analyses

One-color comparative hybridization was performed using Whole Mouse Genome Oligo 4_44K Microarray (G2519F-014868, Agilent Technologies, Les Ulis, France) to compare gene expression profiling. cRNAs were synthesized from 100 ng total RNA, labeled using a Quick Amp Labeling Kit (Agilent Technologies), and hybridized on microarrays at 65 °C for 17 h. Raw hybridization data, evaluated on every probe 5 µm-sized array, using a DNA microarray scanner G2565CA (Agilent Technologies), were extracted with Feature Extraction Software 10.5.1.1 (Agilent Technologies), then transferred to Genespring^®^ (GX 12.6 software, Agilent Technologies) for data processing (normalization) and data mining (Figure 10B).

### 4.4. Genespring Analysis and Generation of Control and PAE Placenta–Cortex Signatures

Data were normalized by the 75th percentile and in each array, outlier spots and those exhibiting heterogeneous signals or those that were not above the background were discarded. Data scale-up was performed at “baseline to median of all samples”. Volcano-plots were supported by the statistical significance of differential gene expression (fold change cut-off = 2) performed with Student’s *t*-test with Benjamini–Hochberg correction for multiple testing to check the False Discovery Rate (FDR; *p* value < 0.05). Genespring analysis lead to the generation of four lists of genes: (1) transcripts over-expressed (at least twofold) in the cortex versus placenta in control mice, (2) transcripts over-expressed (at least twofold) in the cortex versus placenta in PAE mice, (3) transcripts under-expressed (at least twofold) in the cortex versus placenta in control mice and (4) transcripts under-expressed (at least twofold) in the cortex versus placenta in PAE mice (Figure 10C).

### 4.5. Excel Comparison of Control and PAE Placenta–Cortex Signatures

For each independent experiment, the two control lists (transcripts at least 2× over- or under-expressed in cortex versus placenta) generated by the *Genespring* analysis were downloaded in the Excel software and filtered as follows: (*i*) removal of genes notified Riken or unknown, (*ii*) selection of genes found over- or under-expressed in cortex versus placenta in at least 3 out of 4 independent experiments (statistical threshold of 0.75). A similar filtration was performed for the two PAE lists (transcripts over- or under-expressed in cortex versus placenta in PAE mice) generated by the Genespring analysis (Figure 10E,F). Comparison of the two brain–placenta signatures lead to the generation of six lists of genes: (1) genes over-expressed (at least twofold) in the cortex versus placenta and only found in the control signature, (2) genes over-expressed (at least twofold) in the cortex versus placenta and only found in the PAE signature, (3) genes under-expressed (at least twofold) in the cortex versus placenta and only found in the control signature, (4) genes under-expressed (at least twofold) in the cortex versus placenta and only found in the PAE signature, (5) genes common in the two placenta–cortex signatures (control, PAE) and up-regulated by at least 40% after PAE and (6) genes common in the two placenta–cortex signatures (control, PAE) and down-regulated by at least 40% after PAE. A 40% cut-off was chosen to ensure high robustness and stringency of the PAE effects. This range is compatible with the range of variations previously reported regarding the effect of PAE on genes from the VEGF family [9,35]. Altogether, these lists constituted the placenta–cortex signature of genes dysregulated by PAE.

### 4.6. Gene Ontology Analysis

In order to characterize biological function relationships between genes of the placenta–cortex signature dysregulated by PEA, a Gene Ontology analysis was performed (http://geneontology.org (accessed on 20 July 2021)). Based on previous data supporting that PAE impairs vascular development [9], a GO analysis focused on vascular development was performed (Figure 10G). GO terms belonging to the “circulatory system development” [GO:0072358] branch of the GO organizational tree were extracted using the GO browser tool. Genes from the list “placenta–cortex signature dysregulated by PAE” were screened through GO terms related to the vascular development [GO:0001944]. The bioinformatic analysis generated the list of genes belonging to the placenta–cortex signature, dysregulated by PAE and related to vascular development (Figure 10G).

### 4.7. String Analysis

Genes characterized by (*i*) a differential expression between the control and PAE placenta–cortex signatures and (*ii*) an involvement in vascular development were submitted to a String network analysis in order to research functional clusters and enriched protein–protein interactions (PPI; https://string-db.org/ (accessed on 16 January 2023)). Basic settings of the analysis consisted of identifying edges for proteins with functional and physical associations [45,46]. The bioinformatic sources used to determine PPI were based on the following criteria: text mining, databases, experiments, co-expression, neighborhood, gene fusion and co-occurrence. Confidence of PPI was visualized according to line thickness reflecting interaction scores: from the thinnest to the thickest lines, low (0.15), medium (0.4), high (0.7) and highest (0.9) confidence. The affiliation of PPI to the same functional cluster was notified by the same node color. Statistical analysis provided a PPI enrichment *p*-value which indicated that proteins have more interactions among themselves than would be expected for a random set of proteins of the same size and degree distribution drawn from the genome. Such an enrichment indicated that the proteins were at least partially biologically connected, as a group (Figure 10H). Because they could be indicative of a placenta–cortex communication, particular attention was paid to proteins belonging to ligand/receptor couples.

### 4.8. PANTHER GO Analysis

GO analysis was performed on each separate cluster list using protein analysis through evolutionary relationships (PANTHER) version 17.0 software (http://www.pantherdb.org (accessed on 17 January 2023)) and *p*-values less than 0.05 were considered statistically significant [26]. The GO list classification was achieved according to the Gene Ontology “protein class”. The analysis was followed by an over-representation test for the identification of enriched terms in GO “Biological process” (Figure 10I). Statistical significance was calculated based on Fisher’s exact test and adjusted using the false discovery rate (FDR) for correction of multiple tests.

### 4.9. Western Blot Validation

GD20 placentas and paired E20 cortices were homogenized in ice-cold lysis buffer (Cell Signaling Technology, Danvers, MA, USA). The homogenates were centrifuged (18,000× *g*; 20 min), and the supernatants were used for Western blotting (Figure 10J). Fifty micrograms of protein extracts from cortical and placental samples, as determined by the Bradford assay, was denatured at 100 °C for 5 min in Laemmli buffer (Tris-HCl 0.5 M; pH 6.8; SDS 8%; bromophenol blue 0.5%; glycerol 10%; β-mercaptoethanol 10%) and loaded onto a 10% SDS-polyacrylamide gel. After electrophoresis, proteins were transferred to a nitrocellulose or PVDF membrane. Depending on the considered primary antibody, the membrane was incubated with different blocking solution (1× Tris buffer saline (TBS); 0.05% TWEEN 20; 5% nonfat dry milk or 1× TBS; 0.05% TWEEN 20; 5% BSA) at room temperature for 1.5 h. Afterwards, membranes were incubated overnight with primary antibodies (Supplementary Table S22). After incubation with the corresponding secondary antibodies coupled to horseradish peroxidase, proteins were visualized using an enhanced chemiluminescence ECL Plus immunoblotting detection system (Amersham Biosciences Europe GmbH, Freiburg, Germany). The intensity of the immunoreactive bands was quantified using a blot analysis system (Bio-Rad Laboratories, Marne la coquette, France) and β-actin was used as a loading control. Commercial markers (Seeblue prestained standard, Invitrogen, Carlsbad, CA, USA) were used as molecular weight standards.

### 4.10. Description of Human Cases

Human alcohol case (BH19.754): The first case concerns a neonate born at 36 WG and 5 days with morphological facial particularities characterized by a domed philtrum and thin lips, giving rise to reasonable suspicion of the presence of fetal alcohol syndrome. The mother consumed alcohol at the rate of one bottle per day until 16 WG. She also presented with cholestasis during pregnancy. The placenta was fixed in formalin. Then, systematic samples were taken, embedded in paraffin and 6 µm sections were examined after hematoxylin–eosin–saffron (HES) staining. On histology, the placenta was of normal configuration, and its weight was between the 25th and the 50th percentile. There were placental changes related to chronic fetal distress. Human control case (BH19.6192): The next case concerns a neonate born at 38 WG and 6 days without particular anomaly. No alcohol consumption was reported by the mother or the entourage or clinically suspected. The placenta was fixed in formalin. Then, systematic samples were taken, embedded in paraffin and examined after HES staining. On histology, the placenta was of normal configuration, the weight of which was between the 25th and the 50th percentile. No anomaly was found.

### 4.11. Immunohistochemistry in Human Placentas and Line-Scan Analysis

Immunohistochemical visualization of Agt-positive cells was carried out on six-micrometer sections obtained from paraffin-embedded material according to standardized protocols using an Agt-specific antibody (Supplementary Table S22). Before incubation, slices were submitted to deparaffinization which consisted of immersing the slides in successive baths of xylene (100%), ethanol (100–50%) and deionized water. Antigen retrieval was processed by microwave pretreatment in boiling 10 mM sodium citrate buffer (pH 6.0) for 10 min. Incubation of the primary antibody was performed for 60 min at room temperature using the Ventana Benchmark XT system. Slides were then processed with the Ultraview Universal DAB detection kit (Ventana). After 20×, 40× and 63× magnification acquisitions using a Leica Thunder Imaging System, high-resolution images were acquired in tiff format. Afterwards, Agt-positive intensity profiles were created based on villi thickness using the linescan tool of the Metamorph^®^ software (Roper Scientific, Downingtown, PA, USA). After background correction, the area under the curve was calculated in the syncytiotrophoblast, giving access to a relative comparison with the intravillous space.

### 4.12. Statistical Analyses

Statistical analysis of the whole mouse genome microarrays data for placentas and cortices from control and PAE groups was performed using GeneSpring GX (Agilent, Santa Clara, CA, USA). Statistical analysis of predictive PPI was performed using the *String Network* analysis database (https://string-db.org (accessed on 16 January 2023)). Statistical significance was calculated as a PPI enrichment *p*-value representative of the number of edges in the network versus the expected number of edges in a random set of proteins. *p*-values were corrected for multiple testing using the Benjamini–Hochberg test. The K-means algorithm was used to cluster proteins into 4 groups. Statistical analysis of enriched biological processes in each identified cluster was performed using PANTHER GO analysis. Statistical significance was calculated according to Fisher’s exact test and adjusted using the false discovery rate (FDR) for correction of multiple tests. Statistical analysis of protein expression validation by Western blot was performed using the two-way ANOVA test followed by the Bonferroni post-test (when females and males were separately considered) and the Mann–Whitney test (when females and males were pooled). Analysis of ligand–receptor correlation between the placenta and cortex was performed using the Pearson correlation test. The statistical data for each experiment are detailed in Supplementary Table S1.

## Figures and Tables

**Table 1:** PANTHER classification of protein clusters by biological process. Annotation and functional classification of the identified proteins in each cluster was performed using the PANTHER Overrepresentation test. Using this system, the proteins were assigned to Gene Ontology domains [biological process]. The listed functional categories are successfully meeting *p* < 0.05 for Fisher’s test adjusted by the false discovery rate.

		Clusters		
		Transcriptional Regulation ●	Intercellular Communication ●	Structural Proteins and Regulation ●
Biological process: Panther overrepresentation test (Fisher, False Discovery Rate *p* < 0.05)	Cardiac muscle tissue development	2: Sgcg, Sgcd FDR = 5.62 × 10^−3^		
	Notch signaling pathway	4: Llgl2, Notch1, Dll1, Hey2 FDR = 1.14 × 10^−5^		
	Morphogenesis of an epithelium	2: Tbx20, Vangl2 FDR = 1.04 × 10^−2^		
	Cell fate specification	2: Tbx20, Tbx1 FDR = 1.63 × 10^−2^		
	Embryonic organ development	2: Tbx20, Gata3 FDR = 1.81 × 10^−2^		
	Heart contraction	2: Sgcg, Sgcd FDR = 2.04 × 10^−2^		
	Embryonic morphogenesis	2: Tbx20, Msx1 FDR = 2.88 × 10^−2^		
	Pattern specification process	3: Acvr1, Tbx20, Hey2 FDR = 2.41 × 10^−3^		
	Regulation of cell differentiation	3: Gata3, Runx1, Hey2 FDR = 1.71 × 10^−2^		
	Regulation of multicellular organismal process	4: Tbx1, Gata3, Cxcl17, Hey2 FDR = 1.70 × 10^−2^		
	Nervous system development	5: Zic3, Notch1, Grhl2, Runx1, Hey2 FDR = 1.05 × 10^−2^		
	Regulation of transcription by RNA polymerase II	13: Tbx20, Zic3, Tbx1, Msx1, Tcf7l2, Grhl2, Gata3, Foxp1, Hmgb1, Sall4, Arid1a, Runx1, Hey2 FDR = 5.07 × 10^−7^		
	Regulation of angiogenesis		2: Serpinf1, Vegfc FDR = 3.85 × 10^−2^	
	Angiogenesis		3: Serpinf1, Vegfc, Angpt1 FDR = 2.11 × 10^−2^	
	Axon guidance		4: Plxnd1, Efna3, Ephb1, Notch3 FDR = 9.67 × 10^−3^	
	Transmembrane receptor protein tyrosine kinase signaling pathway		8: Tgfa, Lepr, Efna3, Fgf15, Vegfc, Angpt1, Fgf18, Angptl1 FDR = 6.27 × 10^−5^	
	Regulation of cell migration		4: Plxnd1, Fgf15, Vegfc, Fgf18 FDR = 1.38 × 10^−2^	
	Positive regulation of cell population proliferation		4: Tgfa, Fgf15, Vegfc, Fgf18 FDR = 1.51 × 10^−2^	
	Cellular response to growth factor stimulus		4: Fgf15, Vegfc, Tgfbr1, Fgf18 FDR = 2.07 × 10^−2^	
	Positive regulation of protein phosphorylation		4: Tgfa, Fgf15, Vegfc, Fgf18 FDR = 2.27 × 10^−2^	
	Cellular response to endogenous stimulus		5: Rarb, Lepr, Fgf15, Tgfbr1, Fgf18 FDR = 1.63 × 10^−2^	
	Cell migration		5: Itgb8, Plxnd1, Fgf15, Vegfc, Fgf18 FDR = 2.92 × 10^−2^	
	Protein phosphorylation		7: Tgfa, Fgf15, Vegfc, Tgfbr1, Prkcb, Fgf18, Cdk1 FDR = 9.44 × 10^−3^	
	Negative regulation of cellular process		10: Rarb, Reck, Ddit3, Lepr, Plxnd1, Nos3, Serpinf1, Dusp9, Cdk1, Aplnr FDR = 1.41 × 10^−2^	
	Muscle cell differentiation			3: Neb, Myl2, Actn2 FDR = 2.65 × 10^−2^

**Table 2:** Candidate ligand/receptor couples potentially dysregulated by in utero alcohol exposure. Filtration has been applied to select proteins that belong to ligand/receptor couples. The table has been built to represent ligands (placental or cortical) on the left side and their corresponding receptors (cortical or placental) on the right. Ligands or receptors identified from the bioinformatic analysis are indicated in bold characters, with the information concerning the condition in which the protein was detected (Ctrl or Ethanol or both). Proteins that do not belong to the 107 identified candidates are indicated in light characters, with a mention of the amplitude and robustness of alcohol effect on their brain/placenta ratio (+/−40%, in x out of four replicates).

Potentially Dysregulated Ligand/Receptor Couples			
**Placental ligand**	**Alcohol effect** **on cortex/placenta ratio:**	**Cortical receptor**	**Alcohol effect** **on cortex/placenta ratio:**
**Agt—Angiotensinogen**	**Down** **≥** **×2 in Ctrl**	**Agtr2—Type-2 angiotensinogen II receptor**	**Up** **≥** **×2 in Ctrl**
**Angptl2—Angiopoietin-related protein 2**	**Down** **≥** **×2 in EtOH**	CD146 (MCAM)	+40% (1/4)
**Cxcl17—Chemokine (c-x-c motif) ligand 17**	**Down** **≥** **×2 in EtOH**	GPR35	+40% (1/4)
**Tnf—Tumor necrosis factor**	**Down** **≥×** **2 in EtOH**	TNFR (1a)	Multiple
		TNFRB	40% (1/4)
Activin (inh A and B)	no effect	**Acvr1—Activin receptor type-1**	**Up** **≥** **×2 in Ctrl**
Jagged1		**Notch1—Neurogenic locus homolog protein 1**	**Up** **≥×** **2 in Ctrl**
Jagged2		**Notch3—Neurogenic locus homolog protein 3**	**Up** **≥×** **2 in EtOH**
Dll1	−40% (1/4)	**Hey2**	**Up** **≥×** **2 in EtOH**
Apelin	+40% (2/4)	**Aplnr—Apelin receptor**	**Up** **≥** **×2 in EtOH**
UNC5D	+40% (3/4)	**Flrt2—Leucine-rich repeat transmembrane protein**	**Up** **≥** **×2 in EtOH**
Fibronectin	+40% (3/4) (type III)	**Itgb8—Integrin alpha-V:beta-8**	**Up** **≥** **×2 in EtOH**
SEMA4A	+40% (2/4)	**Plxnd1—Plexin-D1**	**Up** **≥×** **2 in EtOH**
SEMA3A	−40% (1/4)		
SEMA3C	no effect		
SEMA3E	+40% (3/4)		
retinoic acid	na	**Rarb—Retinoic acid receptor beta**	**Up** **≥** **×2 in EtOH**
TGFB1	+40% (1/4)	**Tgfbr1—Transforming growth factor, beta receptor I**	**Up** **≥×** **2 in EtOH**
TGFB2	+40% (1/4)		
TGFB3	+40% (2/4)		
EphrinB1	+40% (1/4)	**Ephb1—Ephrin type-B receptor 1**	**Up** **≥×** **2 in Both**
EphrinB2	no effect		
EphrinB3	+40% (2/4)		
**Cortical ligand**	**Alcohol effect** **on cortex/placenta ratio:**	**Placental receptor**	**Alcohol effect** **on cortex/placenta ratio:**
Leptin	+40% (1/4)	**Lepr—Leptin receptor**	**Down** **≥** **×2 in Ctrl**
**Dll1—Delta-like protein 1**	**Up** **≥** **×2 in Ctrl**	Notch2	+40% (1/4)
**Efna3—Ephrin-A3**	**Up** **≥** **×2 in Ctrl**	Ephrin-R	no effect
**Mdk—Midkine**	**Up** **≥** **×2 in Ctrl**	ALK	no effect
**Egf—Pro-epidermal growth factor**	**Up** **≥** **×2 in EtOH**	EgfR	+40% (2/4)
**Fgf18—Fibroblast growth factor 18**	**Up** **≥×** **2 in EtOH**	FGFR3	+40% (3/4)
**Fgf15—Fibroblast growth factor 15**	**Up** **≥×** **2 in Both**	FGFR4	+40% (2/4)
**Tgfa—Protransforming growth factor alpha**	**Up** **≥** **×2 in EtOH**	EGFR	+40% (2/4)
**Vegfc—Vascular endothelial growth factor C**	**Up** **≥×** **2 in EtOH**	VEGFR1 (flt1)	+40% (1/4)
		VEGFR2	
		VEGFR3 (flt4)	+40% (2/4)
**Angpt1—Angiopoietin-1**	**Up** **≥** **×2 in Both**	TIE2 (TEK)	+40% (1/4)
**Igf1—Insulin-like growth factor I**	**Up** **≥** **×2 in Both**	IGF-1R	+40% (1/4)

## Data Availability

The data discussed in this publication have been deposited in NCBI’s Gene Expression Omnibus [47] and are accessible through GEO Series accession number GSE241836 (https://www.ncbi.nlm.nih.gov/geo/query/acc.cgi?acc=GSE241836, accessed on 27 August 2023).

## Supplementary Materials

The following supporting information can be downloaded at: https://www.mdpi.com/article/10.3390/ijms241713484/s1.
