# Supplementary material for: Prenatal Alcohol Exposure Impairs the Placenta–Cortex Transcriptomic Signature, Leading to Dysregulation of Angiogenic Pathways
Source: Int J Mol Sci. 2023 Aug 30;24(17):13484. doi: 10.3390/ijms241713484 (PMC10488081; doi:10.3390/ijms241713484)
Supplement: Supplementary file 1 [file ijms-24-13484-s001.zip › Supplementary Materials.pdf]

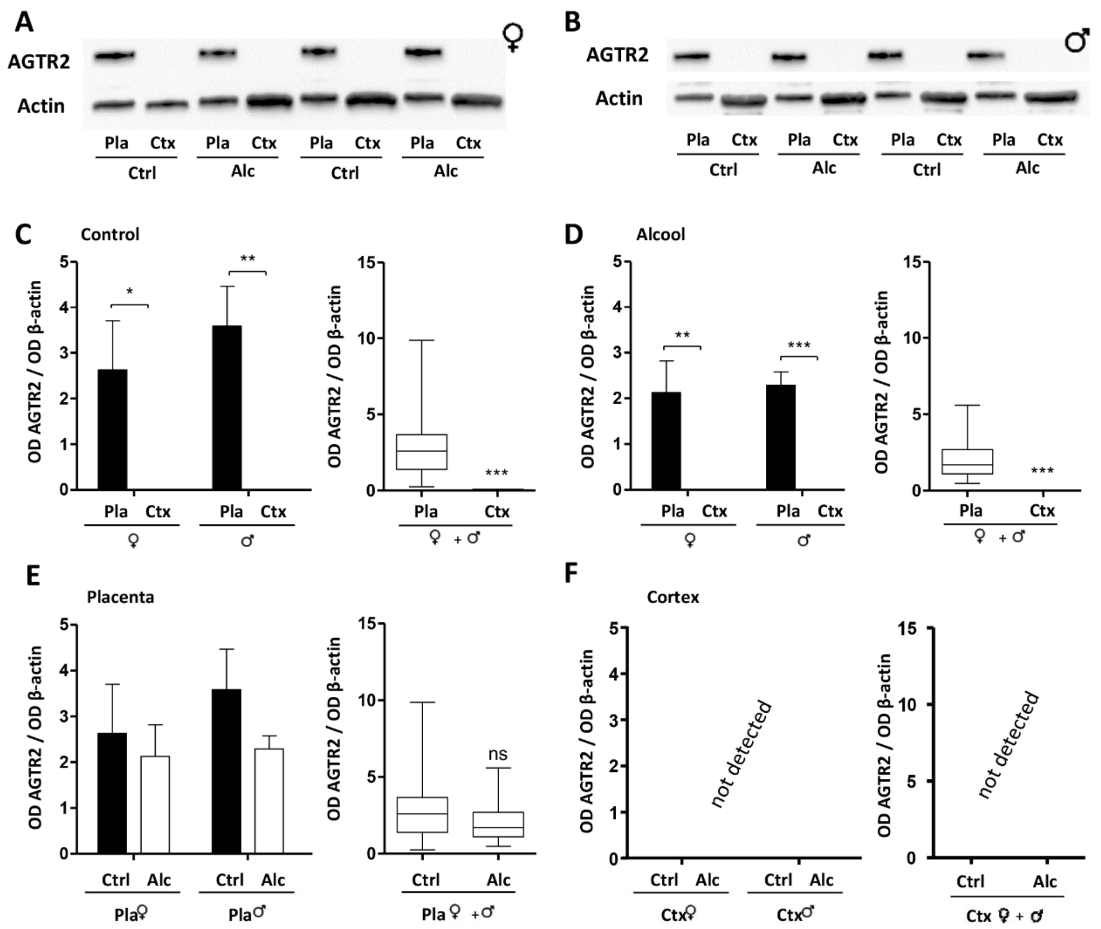

Supplementary Figure 1, Sautreuil et al.

Figure S1. Protein validation of AGTR2 expression in paired placenta/cortex extracts from control and alcohol-exposed mice. **(A,B)** Western blots visualizing the relative expression of AGTR2 in placentas and paired cortices of female **(A)** and male **(B)** fetuses from control and alcohol-exposed mice. **(C)** Quantification of the relative expression of AGTR2 in the control group. **(D)** Quantification of the relative expression of AGTR2 in the PAE group. **(E)** Comparison of AGTR2 expression in the placenta of control and PAE mice. **(F)** Comparison of AGTR2 expression in the cortex of control and PAE fetuses at E20. When compared to placenta, AGTR2 were not detected in the fetal cortex.

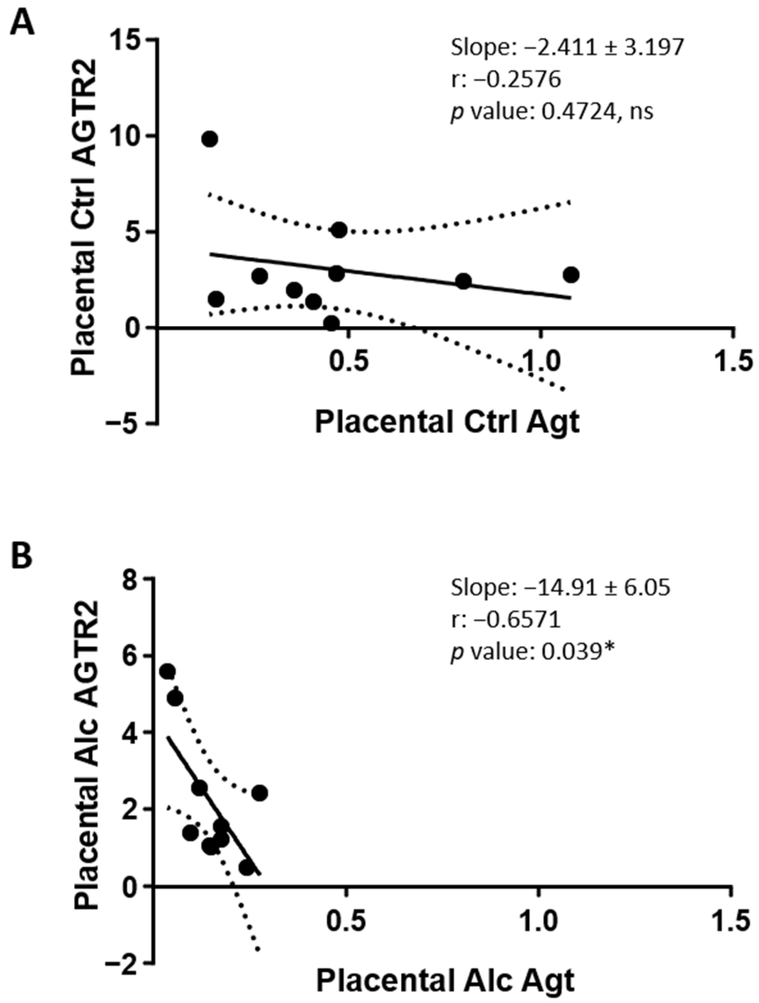

Figure S2. Pearson correlation analysis between placental expression of angiotensinogen and placental expression of AT2 receptors. **(A)** Graph visualizing the paired expression of placental

AGT and AGTR2 receptors in the control group. **(B)** Graph visualizing the paired expression of placental AGT and AGTR2 in the PAE group.

Table S1. Statistical analysis. For each experiment, the tests used, the number of independent experiments and statistic data including *p* values are detailed.

Table S2. Detailed list of genes under- or over-expressed between the two organs in control groups. Among the 26977 genes inversely expressed in cortex versus placenta in the control group, 12304 entities are significantly up or down expressed ($\alpha<0.05$) by a factor of at least 2. These entities are distributed between 6066 ≥2× down and 6238 ≥2× up expressed in the cortex versus placenta.

Table S3. Detailed list of genes under- or over-expressed between the two organs in ethanol groups. Among the 27262 genes inversely expressed in cortex versus placenta in the ethanol group, 12910 entities are significantly up or down expressed ($\alpha<0.05$) by a factor of at least 2. These entities are distributed between 6326 ≥2× down and 6584 ≥2× up expressed in the cortex versus placenta.

Table S4. Detailed list of the control- signature 2-fold over-expressed specific genes resulting from the Genespring analysis. A Venn analysis of the entities ≥2× up expressed in the cortex versus placenta (6238 in control condition and 6584 in ethanol) reveals 402 entities specifically present in the control group. For each entity, the ratio cortex/placenta is calculated (columns N-Q). For each independent microarray, the result is highlighted in green if the ratio is ≥2. The score (column R) indicates in how many experiments the cortex/placenta ratio is above 2. Green-highlighted cells in column R represent entities for whom cortex/placenta ratios are

higher than 2 in at least 3 out of 4 independent experiments (Formula used: =NB.IF(N5:Q5;">=2")). A color code is applied on the "Gene symbol" column (S): yellow, duplicates; brown, RIKENS; grey: unknown genes.

Table S5. Detailed list of the control- signature 2-fold under-expressed specific genes resulting from the Genespring analysis. A Venn analysis of the entities ≥2× down expressed in the cortex versus placenta (6066 in control condition and 6326 in ethanol) reveals 493 entities specifically present in the control group. For each entity, the ratio cortex/placenta is calculated (columns N-Q). For each independent microarray, the result is highlighted in red if the ratio is ≤0.5. The score (column R) indicates in how many experiments the cortex/placenta ratio is below 0.5. Red-highlighted cells in column R represent entities for whom cortex/placenta ratios are lower than 0.5 in at least 3 out of 4 independent experiments (Formula used: =NB.IF(N5:Q5;"<=0.5")). A color code is applied on the "Gene symbol" column (S): yellow, duplicates; brown, RIKENS; grey: unknown genes.

Table S6. Detailed list of the ethanol- signature 2-fold over-expressed specific genes resulting from the Genespring analysis. A Venn analysis of the entities ≥2× up expressed in the cortex versus placenta (6238 in control condition and 6584 in ethanol) reveals 748 entities specifically present in the ethanol group. For each entity, the ratio cortex/placenta is calculated (columns N-Q). For each independent microarray, the result is highlighted in green if the ratio is ≥2. The score (column R) indicates in how many experiments the cortex/placenta ratio is above 2. Green-highlighted cells in column R represent entities for whom cortex/placenta ratios are higher than 2 in at least 3 out of 4 independent experiments (Formula used: =NB.IF(N5:Q5;">=2")). A color code is applied on the "Gene symbol" column (S): yellow, duplicates; brown, RIKENS; grey: unknown genes.

Table S7. Detailed list of the ethanol- signature 2-fold under-expressed specific genes resulting from the Genespring analysis. A Venn analysis of the entities ≥2× down expressed in the cortex versus placenta (6066 in control condition and 6326 in ethanol) reveals 753 entities specifically present in the ethanol group. For each entity, the ratio cortex/placenta is calculated (columns N-Q). For each independent microarray, the result is highlighted in red if the ratio is ≤0.5. The score (column R) indicates in how many experiments the cortex/placenta ratio is below 0.5. Red-highlighted cells in column R represent entities for whom cortex/placenta ratios are lower than 0.5 in at least 3 out of 4 independent experiments (Formula used: =NB.IF(N5:Q5;"<=0.5")). A color code is applied on the "Gene symbol" column (S): yellow, duplicates; brown, RIKENS; grey: unknown genes.

Table S8. Results of the filtrations applied to the list of 402 entities specifically 2-fold over-expressed in control signature. After removal of duplicates (−9 entities), RIKEN sequences (−14 entities), and unidentified genes (−42 entities), the remaining 312 entities are detailed.

Table S9. Results of the filtrations applied to the list of 493 entities specifically 2-fold under-expressed in control signature. After removal of duplicates (−13 entities), RIKEN sequences (−20 entities), and unidentified genes (−58 entities), the remaining 113 entities are detailed.

Table S10. Results of the filtrations applied to the list of 748 entities specifically 2-fold over-expressed in ethanol signature. After removal of duplicates (−23 entities), RIKEN sequences (−45 entities), and unidentified genes (−55 entities), the remaining 610 entities are detailed.

Table S11. Results of the filtrations applied to the list of 753 entities specifically 2-fold under-expressed in ethanol signature. After removal of duplicates (−30 entities), RIKEN sequences (−47 entities), and unidentified genes (−48 entities), the remaining 213 entities are detailed.

Table S12. Detailed list of genes over-expressed between the two organs in both control and ethanol groups. The Genespring analysis revealed 5836 entities present simultaneously among the 6238 Up ≥2× control and the 6584 Up ≥2× ethanol. Columns B to I represent the microarray signals for cortex and placenta, under ethanol treatment (4 replicates). Columns J to Q represent the microarray signals for cortex and placenta, under control condition (4 replicates). Columns R to Y represent the ratio cortex/placenta for each measure (ie: R5=B5/F5). Columns Z to AC represent the effect of ethanol treatment on the cortex/placenta ratio for each replicate. For each microarray, the cortex/placenta ratio after alcohol is expressed as a percentage of its corresponding ratio in control condition (ie: Z5=R5/V5). A score is calculated to select entities whose ratio is modified by ≥ 40% under ethanol treatment in at least 3 out of 4 experiments (columns AD and AE). A color code is applied on Gene symbols (column AF) to highlight: Rikken sequences in brown, Duplicate entities in yellow and unknown entities in grey.

Table S13. Detailed list of genes under-expressed between the two organs in both control and ethanol groups. The Genespring analysis revealed 5573 entities present simultaneously among the 6066 Down ≥2× control and the 6326 Down ≥2× ethanol. Columns B to I represent the microarray signals for cortex and placenta, under ethanol treatment (4 replicates). Columns J to Q represent the microarray signals for cortex and placenta, under control condition (4 replicates). Columns R to Y represent the ratio cortex/placenta for each measure (ie: R5=B5/F5). Columns Z to AC represent the effect of ethanol treatment on the cortex/placenta ratio for each replicate. For each microarray, the cortex/placenta ratio after alcohol is expressed

as a percentage of its corresponding ratio in control condition (ie: Z5=R5/V5). A score is calculated to select entities whose ratio is modified by ≥ 40% under ethanol treatment in at least 3 out of 4 experiments (columns AD and AE). A color code is applied on Gene symbols (column AF) to highlight: Rikken sequences in brown, Duplicate entities in yellow and unknown entities in grey.

Table S14. Results of the filtrations applied to the list of 5836 entities simultaneously 2-fold over-expressed in both control and ethanol signatures. After removal of duplicates (−964 entities), RIKEN sequences (−247 entities), and unidentified genes (−259 entities), the remaining 183 entities are detailed.

Table S15. Results of the filtrations applied to the list of 5573 entities simultaneously 2-fold under-expressed in both control and ethanol signatures. After removal of duplicates (−783 entities), RIKEN sequences (−150 entities), and unidentified genes (−272 entities), the remaining 176 entities are detailed.

Table S16. List of GO terms resulting from the analysis of Gene Ontology biological processes, filtered under the keyword criteria "angiogenesis" OR "Vessel". For each GO term category, are indicated : the number of genes found in mouse genome (reference list; column B), the number of genes found in the upload (filtered genes 2× up or down in ctrl or EtOH or both; column C), and a calculation of fold enrichment (columns D-F).

Table S17. Comparison between genes lists dysregulated by PAE in either cortex or placenta, and "Vascular development" GO terms. The four filtered lists (Up/Down, Ctrl/ethanol; Supplementary tables 8-11) are compared with the list obtained after interrogating Gene

Ontology with the terms "Vascular Development" (column A). The following formula is used to select positive matches which are highlighted in green: =IF(ISNA(VLOOKUP(C3;$A$3:$A$3709;1;0));"Absent";"Present").

Table S18. Comparison between genes lists dysregulated by PAE in both cortex and placenta, and "Vascular development" GO terms. The two filtered lists (Common Up/Common Down; Supplementary tables 14-15) are compared with the list obtained after interrogating Gene Ontology with the terms "Vascular Development" (column A). The following formula is used to select positive matches which are highlighted in green: =IF(ISNA(VLOOKUP(C3;$A$3:$A$3709;1;0));"Absent";"Present").

Table S19. Detailed arborescence of biological processes provided by the PANTHER analysis of the red cluster. The list containing 28 entities that constitute the red cluster were submitted to calculation of fold enrichment among mouse genome GO Biological processes.

Table S20. Detailed arborescence of biological processes provided by the PANTHER analysis of the green cluster. The list containing 34 entities that constitute the green cluster were submitted to calculation of fold enrichment among mouse genome GO Biological processes.

Table S21. Detailed arborescence of biological processes provided by the PANTHER analysis of the blue cluster. The list containing 20 entities that constitute the blue cluster were submitted to calculation of fold enrichment among mouse genome GO Biological processes.

Table S22. Primary and secondary antibodies used for Western blot and immunohistochemistry experiments.
