# Supplementary material for: Prenatal Alcohol Exposure Impairs the Placenta–Cortex Transcriptomic Signature, Leading to Dysregulation of Angiogenic Pathways
Source: Int J Mol Sci. 2023 Aug 30;24(17):13484. doi: 10.3390/ijms241713484 (PMC10488081; doi:10.3390/ijms241713484)
Supplement: Supplementary file 1 [file ijms-24-13484-s001.zip › Supplementary Table S1.pdf]

Supplementary Table S1. Statistical analysis. For each experiment, the tests used, the number of independent experiments and statistic data including *p* values are detailed.

Experiments	Test	n	***p* value**<br>*$p<0.05$; **$p<0.01$; ***$p<0.001$; ****$p<0.0001$
**Figure 1 Whole mouse genome microarrays of placentas and cortices from control and PAE groups**	GeneSpring statistical analysis	n = 4 independent microarray experiments<br>Each experiment consists in 4 groups :<br>Placenta Ctrl<br>Cortex Ctrl<br>Placenta PAE<br>Cortex PAE<br>Each group consists in a pool of 5 placentas Ctrl, 5 cortex Ctrl, 5 placentas PAE, 5 cortex PAE	Volcano-plot were supported by statistical significance of differential gene expression (fold change cut-off = 2) performed with Student's t-test with Benjamini Hochberg correction to check the False Discovery Rate (FDR ; *p* value < 0.05).<br>Hierarchical Clustering: Pearson coefficient metric and complete linkage
**Figure 4 Protein-protein interaction clustering**	String network statistical analysis	List of genes resulting from n = 4 independent experiments.	Number of nodes: 105<br>Number of edges: 228<br>Average node degree: 4.34<br>Avg. local clustering coefficient: 0.447<br>Expected number of edges: 105<br>PPI enrichment p-value: < 1.0×10^{-16}
**Figure 5 Functional classification of protein clusters by protein class**	PANTHER Over-representation test	List of genes resulting from n = 4 independent experiments.	Enriched functional categories meet *p* < 0.05 for Fisher's test.<br>Blue cluster:<br>Intermediate filament binding protein: fold enrichment > 100, FDR 9.23×10^{-3}<br>Intermediate filament: fold enrichment 38.26, FDR 4.40×10^{-2}<br>Cytoskeletal protein: fold enrichment 9.39, FDR 6.25 $\times 10^{-3}$<br>Extracellular matrix structural protein: fold enrichment 40.41, FDR 6.06×10^{-3}<br>Extracellular matrix protein: fold enrichment 19.26, FDR 2.03×10^{-2}

			Actin or actin-binding cytoskeletal protein: fold enrichment 14.27, FDR 8.25 × 10^{-3}<br>Green cluster:<br>Growth factor: fold enrichment 24.37, FDR 2.35× 10^{-5}<br>Intercellular signal molecule: fold enrichment 11.66, FDR 9.25 × 10^{-8}<br>Red cluster:<br>Immunoglobulin transcription factor: fold enrichment 71.42, FDR 8.39 × 10^{-4}<br>DNA-binding transcription factor: fold enrichment 6.04, FDR 5.19 × 10^{-4}<br>Gene-specific transcriptional regulator: fold enrichment 5.58, FDR 5.25 × 10^{-4}
**Figure 6C (left panel)** Western blot analysis of AGT expression in placenta and cortex of females and males in the control group	Two-way ANOVA Bonferroni post-test	n = 6 placenta/cortex pairs from female fetuses<br>n = 6 placenta/cortex pairs from male fetuses	ANOVA<br>Interaction F 0.09324; $p = 0.7633$; ns<br>Gender F 0.001131; $p = 0.9735$; ns<br>Organs F 17.29; $p<0.0005$***<br>Bonferroni's post-test<br>Placenta *vs* Cortex<br>For males $p<0.05$*<br>For females $p<0.05$*
**Figure 6C (right panel)** Western blot analysis of AGT expression in placenta and cortex of pooled females and males from the control group	Mann Whitney test	n = 12 placenta/cortex pairs from female + male fetuses	Placenta *vs* cortex $p<0.0001$****
**Figure 6D (left panel)** Western blot analysis of AGT expression in placenta and cortex of females and males in the PAE group	Two-way ANOVA Bonferroni post-test	n = 6 placenta/cortex pairs from female fetuses<br>n = 6 placenta/cortex pairs from male fetuses	ANOVA<br>Interaction F 0.7816; $p = 0.3872$; ns<br>Gender F 1.842; $p = 0.1898$; ns<br>Organs F 11.98; $p<0.01$**<br>Bonferroni's post-test<br>Placenta *vs* Cortex<br>For males $p<0.05$*<br>For females $p>0.05$; ns

**Figure 6D (right panel)** Western blot analysis of AGT expression in placenta and cortex of pooled females and males from the PAE group	Mann Whitney test	n = 12 placenta/cortex pairs from female + male fetuses	Placenta *vs* cortex *p*<0.0001****
**Figure 6E (left panel)** Western analysis of AGT expression in the placenta of females and males fetuses from control and PAE groups	Two-way ANOVA Bonferroni post-test	n = 6 placentas from female control fetuses<br>n = 6 placentas from male control fetuses<br>n = 6 placentas from female PAE fetuses<br>n = 6 placentas from male PAE fetuses	ANOVA<br>Interaction F 0.4239; *p* = 0.5224; ns<br>Gender F 0.1189; *p* = 0.7339; ns<br>Treatment F 5.405; *p*<0.05*<br>Bonferroni's post-test<br>Control *vs* Alcohol<br>For males *p*>0.05; ns<br>For females *p*>0.05; ns
**Figure 6E (right panel)** Western blot analysis of AGT expression in placentas of pooled females and males from the control and PAE groups	Mann Whitney test	n = 12 placentas from the control group<br>n = 12 placentas from the PAE group	Control *vs* Alcohol *p*<0.05*
**Figure 6F (left panel)** Western analysis of AGT expression in the cortex of females and males fetuses from control and PAE groups	Two-way ANOVA Bonferroni post-test	n = 6 cortices from female control fetuses<br>n = 6 cortices from male control fetuses<br>n = 6 cortices from female PAE fetuses<br>n = 6 cortices from male PAE fetuses	ANOVA<br>Interaction F 0.2203; *p* = 0.8835; ns<br>Gender F 3.673; *p* = 0.0697; ns<br>Treatment F 0.7789; *p* = 0.3880; ns
**Figure 6F (right panel)** Western blot analysis of AGT expression in cortices of pooled females and males from the control and PAE groups	Mann Whitney test	n = 12 placentas from the control group<br>n = 12 placentas from the PAE group	Control *vs* Alcohol *p* = 0.3123; ns

**Figure 7C (left panel)** Western blot analysis of AGTR1 expression in placenta and cortex of females and males in the control group	Two-way ANOVA Bonferroni post-test	n = 8 placenta/cortex pairs from female fetuses<br>n = 8 placenta/cortex pairs from male fetuses	ANOVA<br>Interaction F 0.5249; *p* = 0.4748; ns<br>Gender F 0.4598; *p* = 0.5033; ns<br>Organ F 11.02; *p*<0.01**<br>Bonferroni's post-test<br>Placenta *vs* cortex<br>For males *p*>0.05; ns<br>For females *p*<0.05*
**Figure 7C (right panel)** Western blot analysis of AGTR1 expression in placenta and cortex of pooled females and males from the control group	Mann Whitney test	n = 16 placenta/cortex pairs from female + male fetuses	Placenta *vs* cortex<br>*p*<0.0001****
**Figure 7D (left panel)** Western blot analysis of AGTR1 expression in placenta and cortex of females and males in the PAE group	Two-way ANOVA Bonferroni post-test	n = 8 placenta/cortex pairs from female fetuses<br>n = 8 placenta/cortex pairs from male fetuses	ANOVA<br>Interaction F 0.2933; *p* = 0.5924; ns<br>Gender F 0.7809; *p* = 0.3844; ns<br>Organ F 11.55; *p*<0.01**<br>Bonferroni's post-test<br>Placenta *vs* cortex<br>For males *p*>0.05; ns<br>For females *p*<0.05*
**Figure 7D (right panel)** Western blot analysis of AGTR1 expression in placenta and cortex of pooled females and males from the PAE group	Mann Whitney test	n = 16 placenta/cortex pairs from female + male fetuses	Placenta *vs* cortex<br>*p*<0.0001****
**Figure 7E (left panel)** Western analysis of AGTR1 expression in the placenta of females and males fetuses from control and PAE groups	Two-way ANOVA Bonferroni post-test	n = 8 placentas from female control fetuses<br>n = 8 placentas from male control fetuses<br>n = 8 placentas from female PAE fetuses<br>n = 8 placentas from male PAE fetuses	ANOVA<br>Interaction F 3.466; *p* = 0.0732; ns<br>Gender F 1.935; *p* = 0.1751; ns<br>Treatment F 1.275; *p* = 0.2685; ns

**Figure 7E (right panel)** Western blot analysis of AGTR1 expression in placentas of pooled females and males from the control and PAE groups	Mann Whitney test	n = 16 placentas from the control group<br>n = 16 placentas from the PAE group	Control *vs* Alcohol<br>$p = 0.6647$; ns
**Figure 7F (left panel)** Western analysis of AGTR1 expression in the cortex of females and males fetuses from control and PAE groups	Two-way ANOVA Bonferroni post-test	n = 8 cortices from female control fetuses<br>n = 8 cortices from male control fetuses<br>n = 8 cortices from female PAE fetuses<br>n = 8 cortices from male PAE fetuses	ANOVA<br>Interaction F 0.01039; $p = 0.9195$; ns<br>Gender F 0.9911; $p = 0.3280$; ns<br>Treatment F 0.1580; $p = 0.6940$; ns
**Figure 7F (right panel)** Western blot analysis of AGTR1 expression in cortices of pooled females and males from the control and PAE groups	Mann Whitney test	n = 16 cortices from the control group<br>n = 16 cortices from the PAE group	Control *vs* Alcohol<br>$p = 0.9549$; ns
**Figure 8A** Correlation analysis of placental expression of AGT with cortical expression of AGTR1 in the control group.	Pearson correlation test	n = 10 paired placentas and cortices from the control group	Pearson $r = -0.664$<br>$p = 0.0363$*<br>Slope −1.203 ± 0.4788
**Figure 8B** Correlation analysis of placental expression of AGT with cortical expression of AGTR1 in the PAE group.	Pearson correlation test	n = 10 paired placentas and cortices from the PAE group	Pearson $r = -0.7609$<br>$p = 0.0106$*<br>Slope −4.294 ± 1.295

**Suppl. Figure 1C (left panel)** Western blot analysis of AGTR2 expression in placenta and cortex of females and males in the control group	Two-way ANOVA Bonferroni post-test	n = 8 placenta/cortex pairs from female fetuses<br>n = 8 placenta/cortex pairs from male fetuses	ANOVA<br>Interaction F 0.4721; *p* = 0.4977; ns<br>Gender F 0.4721; *p* = 0.4977; ns<br>Organ F 19.96; *p*<0.0001****<br>Bonferroni's post-test<br>Placenta *vs* Cortex<br>For males *p*<0.01**<br>For females *p*<0.05*
**Suppl. Figure 1C (right panel)** Western blot analysis of AGTR1 expression in placenta and cortex of pooled females and males from the control group	Mann Whitney Test	n = 16 placenta/cortex pairs from female + male fetuses	Placenta *vs* cortex<br>*p*<0.0001****
**Suppl. Figure 1D (left panel)** Western blot analysis of AGTR2 expression in placenta and cortex of females and males in the PAE group	Two-way ANOVA Bonferroni post-test	n = 8 placenta/cortex pairs from female fetuses<br>n = 8 placenta/cortex pairs from male fetuses	ANOVA<br>Interaction F 0.04647; *p* = 0.8309; ns<br>Gender F 0.0447; *p* = 0.8309; ns<br>Organ F 34.92; *p*<0.0001****<br>Bonferroni's post-test<br>Placenta *vs* Cortex<br>For males *p*<0.001***<br>For females *p*<0.01**
**Suppl. Figure 1D (right panel)** Western blot analysis of AGTR2 expression in placenta and cortex of pooled females and males from the PAE group	Mann Whitney Test	n = 16 placenta/cortex pairs from female + male fetuses	Placenta *vs* cortex<br>*p*<0.0001****
**Suppl. Figure 1E (left panel)** Western analysis of AGTR2 expression in the placenta of females and males fetuses from control and PAE groups	Two-way ANOVA Bonferroni post-test	n = 8 placentas from female control fetuses<br>n = 8 placentas from male control fetuses<br>n = 8 placentas from female PAE fetuses<br>n = 8 placentas from male PAE fetuses	ANOVA<br>Interaction F 0.2533; *p* = 0.6187; ns<br>Gender F 0.5003; *p* = 0.4852; ns<br>Treatment F 1.295; *p* = 0.2647; ns

**Suppl. Figure 1E (right panel)** Western blot analysis of AGTR2 expression in placentas of pooled females and males from the control and PAE groups	Mann Whitney Test	n = 16 placentas from the control group<br>n = 16 placentas from the PAE group	Control *vs* Alcohol<br>*p* = 0.300; ns
**Suppl. Figure 1F (left panel)** Western analysis of AGTR2 expression in the cortex of females and males fetuses from control and PAE groups	No statistical analysis (AGTR2 was not detected in the fetal cortex).	n = 8 placentas from female control fetuses<br>n = 8 placentas from male control fetuses<br>n = 8 placentas from female PAE fetuses<br>n = 8 placentas from male PAE fetuses	No data
**Suppl. Figure 1F (right panel)** Western blot analysis of AGTR2 expression in cortices of pooled females and males from the control and PAE groups	No statistical analysis (AGTR2 was not detected in the fetal cortex).	n = 16 placentas from the control group<br>n = 16 placentas from the PAE group	No data
**Suppl. Figure 2A** Correlation analysis of placental expression of AGT with placental expression of AGTR2 in the control group.	Pearson correlation test	n = 10 placentas from the control group	Pearson r = -0.2576<br>p = 0.4724 ; ns<br>Slope = −2.411 ± 3.197
**Suppl. Figure 2B** Correlation analysis of placental expression of AGT with placental expression of AGTR2 in the PAE group.	Pearson correlation test	n = 10 placentas from the PAE group	Pearson r = -0.7609<br>p = 0.039*<br>Slope = −14.91 ± 6.05
