# Supplementary material for: Prenatal Alcohol Exposure Impairs the Placenta–Cortex Transcriptomic Signature, Leading to Dysregulation of Angiogenic Pathways
Source: Int J Mol Sci. 2023 Aug 30;24(17):13484. doi: 10.3390/ijms241713484 (PMC10488081; doi:10.3390/ijms241713484)
Supplement: Supplementary file 1 [file ijms-24-13484-s001.zip › Supplementary Table S22.pdf]

**Supplementary Table S22**. Primary and secondary antibodies used for Western blot and immunohistochemistry experiments.

| **Primary antibody target** | **Trade reference** | **Purified species** | **Supplier** | **Dilution** | **Solution of incubation** |
|---|---|---|---|---|---|
| AGT | NBP1-30027 | Rabbit | Novus Biological | WB 1/1000 IHC 1/200 | BSA (5% TBST)<br><br>1% BSA, 3% Triton X-100 in PBS |
| AGTR1 | AAR-011 | Rabbit | Alomone Labs | WB 1/1000 | Milk (5% TBST) |
| AGTR2 | AAR-012 | Rabbit | Alomone Labs | WB 1/1000 | Milk (5% TBST) |
| **Secondary antibody** | **Trade reference** | **Purified species** | **Supplier** | **Dilution** | **Solution of incubation** |
| Goat anti-Rabbit HRP | 31460 | Goat | ThermoFisher Scientific | WB 1/5000 | Milk (5% TBST) or BSA (5% TBST) |
